# Phylogenetic Classification and Functional Review of Autotransporters

**DOI:** 10.3389/fimmu.2022.921272

**Published:** 2022-07-01

**Authors:** Kaitlin R. Clarke, Lilian Hor, Akila Pilapitiya, Joen Luirink, Jason J. Paxman, Begoña Heras

**Affiliations:** ^1^Department of Biochemistry and Chemistry, La Trobe Institute for Molecular Science, La Trobe University, Melbourne, VIC, Australia; ^2^Department of Molecular Microbiology, Amsterdam Institute of Molecular and Life Sciences (AIMMS), Vrije Universiteit, Amsterdam, Netherlands

**Keywords:** type V secretion system, virulence, bacterial pathogenesis, toxins, adhesins, secreted proteins

## Abstract

Autotransporters are the core component of a molecular nano-machine that delivers cargo proteins across the outer membrane of Gram-negative bacteria. Part of the type V secretion system, this large family of proteins play a central role in controlling bacterial interactions with their environment by promoting adhesion to surfaces, biofilm formation, host colonization and invasion as well as cytotoxicity and immunomodulation. As such, autotransporters are key facilitators of fitness and pathogenesis and enable co-operation or competition with other bacteria. Recent years have witnessed a dramatic increase in the number of autotransporter sequences reported and a steady rise in functional studies, which further link these proteins to multiple virulence phenotypes. In this review we provide an overview of our current knowledge on classical autotransporter proteins, the archetype of this protein superfamily. We also carry out a phylogenetic analysis of their functional domains and present a new classification system for this exquisitely diverse group of bacterial proteins. The sixteen phylogenetic divisions identified establish sensible relationships between well characterized autotransporters and inform structural and functional predictions of uncharacterized proteins, which may guide future research aimed at addressing multiple unanswered aspects in this group of therapeutically important bacterial factors.

## 1 Introduction

Many processes essential for bacterial survival require proteins located extracellularly or at the bacterial surface (1, 2). To facilitate their transport across the cell envelope, bacteria have evolved a diverse range of secretion systems. This includes the secretion of virulence factors that promote bacterial pathogenesis *via* functions such as invasion, adherence, dissemination, and immune evasion (3, 4). Accordingly, these secretion systems are fundamental for bacterial pathogenesis. The most ubiquitous are the Sec and Tat systems, which transport a large variety of proteins across the phospholipid biolayer of the inner membrane (IM) (5). In Gram-negative bacteria, the outer membrane (OM), with phospholipid and lipopolysaccharide leaflets, presents a second barrier to secretion. To overcome the multilayered cell envelope, Gram-negative bacteria possess additional secretion machineries including the chaperone usher system and those classified as type 1 to type 9 secretion systems (T1SS to T9SS) (1, 6). In addition to these established secretion systems, other secretory systems are likely present in Gram-negative bacteria and this list is expected to grow to include further members (7, 8). These systems may directly secrete proteins outside the cell (T1SS and T7SS), traverse multiple membranes and deliver them into the cytoplasm of recipient cells (T3SS, T4SS, T6SS), or transport them across the OM in two steps assisted by the Sec or Tat IM transportation systems (T2SS, T5SS, T8SS, T9SS) (9). Because the periplasm lacks ATP, most of these machineries are large complexes including IM components to access cytoplasmic ATP (10). By comparison, the T5SS does not require ATP and is remarkably simple, typically involving a single dedicated protein (2, 11, 12). This review focuses on the T5SS, alternatively called the autotransporter system reflecting its uniquely simple and energy-efficient transport mechanism.

### 1.1 The T5SS: Autotransporters (ATs)

**Figure 1:**
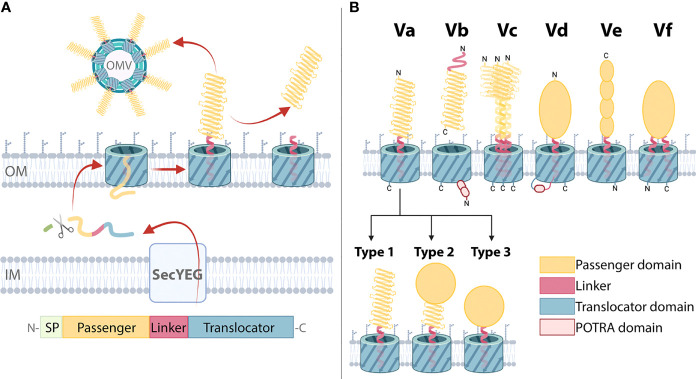
Biogenesis and domain architecture of the type 5 secretion system (T5SS). **(A)** AT secretion mechanism modelled on classical ATs with the following domain organization: The N-terminal signal peptide (SP) is followed by the passenger, linker, and translocator. The SP targets the ATs for inner membrane (IM) secretion via the SecYEG translocon which is subsequently cleaved by a periplasmic peptidase. The translocator inserts into the outer membrane (OM), forming a β-barrel with the α-helical linker spanning its pore. The passenger is translocated to the OM surface where it folds into its tertiary structure. In some ATs, the passenger is cleaved and secreted into the external milieu. Release can also occur through outer membrane vesicles (OMVs). **(B)** T5SS subtypes Va-Vf. Three basic domains (the passenger, linker, and translocator) are present in all T5SS subtypes with variations in topology, domain order, and oligomeric states producing six different subtypes (16–18). These AT classes include: the classical ATs (Va), where the translocator that forms a 12-stranded β-barrel in the outer membrane, and a mostly β-helical passenger, are part of one polypeptide; the two-partner secretion systems (Vb), which are unique because the β-helical passenger is encoded by a separate gene from the translocator, which forms a 16-stranded β-barrel that harbors two polypeptide-transport-associated (POTRA) domains that facilitate the interaction of the passenger and translocators; trimeric ATs (Vc), which require three polypeptides to constitute a full 12-stranded β-barrel translocator to secrete the passengers which includes a coiled-coil stalk and β-helical head regions; patatin-like ATs (Vd), with similar domain architecture to Va but where the translocator is a 16-stranded β-barrel that contains a POTRA domain; inverse ATs (Ve), which comprise an inverted domain organization with an N-terminal signal sequence followed by the translocator, then the linker and a C-terminal passenger; and Hop-family ATs (Vf) possessing an interrupted β-barrel translocator where the passenger is inserted in the loop joining the 1st and second β-strands, and therefore resembling a prolonged loop protruding from the 8-stranded β-barrel. Outer membrane (OM) is indicated. Within classical Va ATs, passengers can adopt various structural configurations: Type 1 passenger structures consist of a β-helix, which may be decorated with functional loops and are connected to the translocator *via* the α-helical linker; in Type 2 structures a catalytic domain is present at the β-helix N-terminus; Type 3 structures lack a β-helix, instead a catalytic domain is directly connected to the translocator *via* the linker. This visual representation of T5SS subtype domain organization is consistent with other reviews (16, 17, 19–22).

The type 5 secretion system (T5SS) is the largest group of secreted proteins in Gram-negative bacteria (13–15). While it encompasses functionally diverse proteins, their journey from cytoplasm to OM is similar (**Figure 1A**) (16, 17). T5SS proteins are termed autotransporters (ATs) because each contains both, secretion machinery (translocator) and functional cargo (passenger) (17). In the cytoplasm, ATs carry an N-terminal signal peptide (SP) for Sec-mediated transport across the IM where the SP is cleaved (23, 24). Periplasmic chaperones keep ATs unfolded until reaching the OM (25–28). The translocator forms a pore in the OM to facilitate the transport of the passenger to the cell surface (29). The passengers are frequently comprised of repetitive secondary structure elements, the sequential folding of which on the bacterial surface may provide a driving force for AT translocation (30–33). The first model of an autotransport mechanism was proposed in 1987 (29) and this has remained an active area of research with several recent reviews on the topic (19, 34, 35). While these basic transport steps are largely consistent with the initial model, later studies revealed the process is not entirely autonomous. Most notably, the barrel assembly machinery (BAM) complex, which catalyzes folding of many OM proteins, is required for insertion of the translocator into the OM and may also facilitate passenger translocation directly (25, 36–39). Significant advances have also been made in our understanding of passenger functions, and these are reviewed in the current work.

While all T5SS members contain both a passenger and translocator, there are variations in their domain arrangement dividing them into subtypes Va to Vf (**Figure 1B**). The Va ATs include, from the N- to C-terminus, a signal peptide, passenger and translocator. The Vc ATs, that include YadA from *Yersinia ssp*. are similar except that their passenger and translocator form trimers, with three ATs forming a single passenger-translocator in the bacterial outer membrane (40, 41). By comparison the Ve ATs represented by intimin from enteropathogenic and enterohaemorrhagic *Escherichia coli* are similar to that of the Va subtype except that their passenger and translocator are switched in position (42). In contrast, the passenger and translocator of Vb ATs such as *Bordetella pertussis* FHA, are expressed as separate proteins. Their translocators include two periplasmic polypeptide-transport-associated (POTRA) domains (20, 43). Similarly, the Vd ATs such as PlpD from *Pseudomonas aeruginosa* and FplA from *Fusobacterium nucleatum* also include a POTRA domain, but only a single POTRA domain exists between the passenger and translocator which are expressed as a single protein (44, 45). Lastly, the type Vf ATs represented by BapA from *Helicobacter pylori* are the most distant subtype, whereby its inclusion into the T5SS is still unclear (18). The likely passenger of the Vf ATs derives from a loop that is part of its putative β-barrel translocator. The Va ATs are the focus of this study, where for clarity, the term ‘AT’ will hereafter refer to this group.

### 1.2 Type Va ATs

ATs are highly diverse outer membrane proteins that are distributed widely throughout Gram-negative bacteria, including the phylum Fusobacteria, the order Chlamydiales and all classes of Proteobacteria (14). However, each AT exhibits a similar domain organization consisting of an N-terminal SP followed by a passenger, linker, and C-terminal translocator (**Figure 1A**) (29, 46, 47).

#### 1.2.1 Translocator: Conserved Sequence, Structure, and Function

Translocators exhibit sequence conservation corresponding to the Pfam entry PF03797 (48) and form β-barrel structures that span the OM and facilitate passenger translocation (14, 47, 49–53). The first translocator crystal structure, NalP from *Neisseria meningitidis*, revealed a monomeric, 12-stranded β-barrel forming a 10 Å by 12.5 Å pore (47). Homologous structures have since been determined for distantly related ATs AIDA-I, Hbp/Tsh, EspP, EstA, NalP, and BrkA (50–54). Along with the observation that chaperones are required for proper secretion, the narrow pore size suggests passengers are unfolded during translocation (19, 27, 36, 47). However, folded passengers may be secreted through a larger pore formed by the translocator together with the BamA insertase (19, 25, 55). Despite this, there are limitations on the complexity of folded regions tolerated (31, 56, 57).

#### 1.2.2 The Linker Domain, Cleavage, and Release

The linker connects the passenger and translocator, where after transport of the passenger to the bacterial surface, the linker forms an α-helix spanning the translocator pore (54). In many cases, the passenger is cleaved from the translocator either within the linker or at a nearby site. Cleavage is catalyzed by separate proteases or by the AT itself *via* its own protease subdomain contained within the passenger, or through an autoproteolytic mechanism within the β-barrel (58–64). Many ATs remain at the bacterial surface, either covalently attached to the translocator or through non-covalent interactions after cleavage (65–68). These ATs influence the surface properties of bacteria such as AIDA-I promoting bacterial aggregation through self-adhesion (65). Other ATs are released into the external milieu to act on targets away from the bacterial surface, for example the passenger of IgA1 protease is proteolytically released and moves away to cleave host immunoglobulins (29). ATs can also be released *via* outer membrane vesicles (OMVs) that pinch off from the OM, for example Vag8 released in OMVs activates and depletes host immune factors away from the bacterial surface (68, 69).

#### 1.2.3 Passenger: Common Structural Themes

Passengers execute the specific function of each AT, and thus show more sequence variation compared to the translocators (49). Despite their sequence and functional diversity, passenger structures are strikingly similar. Most are predicted to include β-solenoid content, with over 90% of published passenger structures comprising a right-handed three-stranded β-helix (70–81). Although the β-helix structure predominates, variations include β-helices with curved or extended sections and the addition of subdomains and loops that protrude out from the β-helix (70–78, 80, 81). The passenger β-helix facilitates multifunctionality as it may directly function as a binding domain specialized to interact with specific host or bacterial factors (70, 71) and can act as a scaffold for catalytic subdomains (72–75, 77, 81). Notably, some ATs lack β-helical structure entirely, for instance, EstA from *P. aeruginosa* is the only published passenger structure comprised of a globular catalytic domain attached directly to the linker (54). Taken together, published AT passenger structures can be divided into three broad types: Type 1, β-helix only; Type 2, globular enzymatic domain supported by a β-helix stalk; Type 3, enzymatic domain without a β-helix (**Figure 1B**). However, given the small proportion of AT structures available the full extent of structural variation within this family remains to be fully uncovered.

### 1.3 Functional Properties of AT Proteins

ATs are multifunctional proteins that contribute to supporting bacterial survival and growth in different environments. Of significance is that many of these functions are virulence traits that enhance bacterial pathogenic potential (14, 82–87). AT passengers exhibit highly varied sequences, consistent with the variety of functions they perform (88). Some examples of the roles executed by ATs include host adhesion, auto-aggregation, biofilm formation, hemagglutination, invasion, intracellular motility, toxicity, and immune evasion, along with enzymatic functions such as protease, lipase, and sialidase activities (16). In many cases, these ATs are expressed by bacterial pathogens where these activities promote disease.

Based on functional properties, some classical AT proteins are classified into four broad groups. These are the serine protease ATs of *Enterobacteriaceae* (SPATEs) (87), subtilisin-like ATs (17), self-associating ATs (SAATs) (89), and GDSL-lipases (90).

SPATEs are a family of secreted AT toxins that cleave a variety of host substrates including fodrin, hemoglobin, mucin and Factor V, among others (91). SPATEs are probably the best-studied group of ATs where several reviews have covered current knowledge about SPATE functions (87, 91–94). The passenger of these ATs incorporates a β-helical scaffold with an N-terminal chymotrypsin-like subdomain corresponding to the S6 serine protease family in the MEROPS database (49, 95). Detailed phylogenetic analysis performed on SPATEs have divided these proteins into Class-1 cytotoxins that degrade intracellular substrates and Class-2 immunomodulators that degrade extracellular substrates (87).

Another group of AT proteases are the subtilisin-like ATs, which may be anchored to the bacterial surface or released into the extracellular environment (96–98). These ATs are predicted to contain a β-helical stalk with an N-terminal subtilisin-like subdomain corresponding to the S8A serine protease family in the MEROPS database (17, 95). Overall, subtilisin-like AT functions are poorly understood, but have been associated with surface maturation of other virulence factors to promote virulence functions like cytotoxicity, aggregation, and hemagglutination (17).

Self-associating ATs (SAATs) are a prominent functional subgroup in the AT superfamily (89). These diverse OM-anchored adhesins are predicted to share β-helix architecture in their passenger, as shown for two canonical SAATs, Ag43 and TibA (71, 80). Although ATs in this group can have different functions, all promote bacterial aggregation and biofilm formation through self-association between passengers on neighboring bacteria (71, 89).

Another class of ATs with catalytic activity are the GDSL-lipase ATs. These ATs lack the archetypal β-helix scaffold found in the majority of ATs (54, 90) and are primarily membrane anchored where they hydrolyze ester bonds in host or bacterial lipids (90). Although their natural substrates are unknown, it is assumed they hydrolyze membrane lipids, where they have been shown to affect host cell lysis, lipid and phosphate metabolism, adhesion, and motility (90).

While the identification and definition of these functional groups has provided an important framework for understanding AT proteins, many ATs have been characterized that do not belong to these established functional group.

## 2 Phylogenetic Classification of AT Proteins

Over the past decades, different groups have devoted considerable effort to the phylogenetic characterization of AT proteins. Henderson, et al. (17) published a landmark phylogenetic analysis of ATs with described phenotypes. This analysis used the sequences of the more conserved AT translocator resulting in the division into 11 subgroups. This enabled comparison and description of the functions within each phylogenetic group and has provided a guiding principle for AT research for the last 18 years. Since this time Celik, et al. (14) using a bioinformatics strategy, presented a large-scale phylogenetic analysis with hundreds of predicted AT passenger sequences, which highlighted the anticipated diversity and widespread distribution of these proteins. Additionally, other phylogenetic analyses have been reported focused on specific AT subgroups (21, 87, 88, 99). With the advent of genome sequencing techniques, the past years have seen a substantial increase in the number of AT sequences reported in public databases along with a steady rise in AT functional characterization, to the point where there is now sufficient data for functional phylogenetic classification studies.

### 2.1 Sequence Alignment of Characterized ATs

In this work we sought to carry out a comprehensive analysis of functionally characterized ATs. Given the passenger of ATs is the region primarily responsible for facilitating the associated bacterial phenotype through its interactions with the host and/or environment, our analysis concentrated on AT passengers alone to gain insights into the functional relationships between ATs.

Functionally characterized ATs were identified from the literature, particularly focusing on previous reviews (16, 17, 19, 94) and by searching published databases (PubMed and Web of Science) using the keywords “autotransporter” and “T5SS”. After eliminating those lacking experimental characterization, 112 ATs were identified from 32 species across 24 genera of Gram-negative bacteria. Proteobacteria accounted for 97 ATs including classes α-proteobacteria (8 ATs), β-proteobacteria (16 ATs), ϵ-proteobacteria (7 ATs), and γ-proteobacteria (66 ATs, including 31 from *E. coli*). Twelve ATs from Chlamydiae and 3 ATs from Fusobacteria are also represented. Full-length amino acid sequences were retrieved from the National Centre of Biotechnology (NCBI) for prediction of the SP, α-helical linker, and translocators using SignalP 4.1 (100), PSIPRED (101), and InterPro (102), respectively. **Table S1** details the accession numbers for all 112 ATs analyzed. Passenger sequences were identified and recorded as the region flanked by the SP and α-helical linker. PSIPRED secondary structure predictions were also used to predict the secondary structure of the passengers. Clustal Omega (103) was used to generate a multiple sequence alignment of the passengers, which demonstrated high diversity within the AT family. Consistent with previous reports (14), we found that passenger lengths were highly varied, ranging from 193 to 3,374 aa with an average of 945 aa (**Supplementary Figure S1**). This diversity of sequence lengths between ATs may have skewed some of the phylogenetic relationships, particularly for very short and very long sequences. A heatmap of pairwise identities (**Supplementary Figure S2**) from the alignment identified 15 high-identity groups, with low identities between the groups, indicating that each group is highly unique.

### 2.2 Functional Phylogenetic Classification of ATs

**Figure 2:**
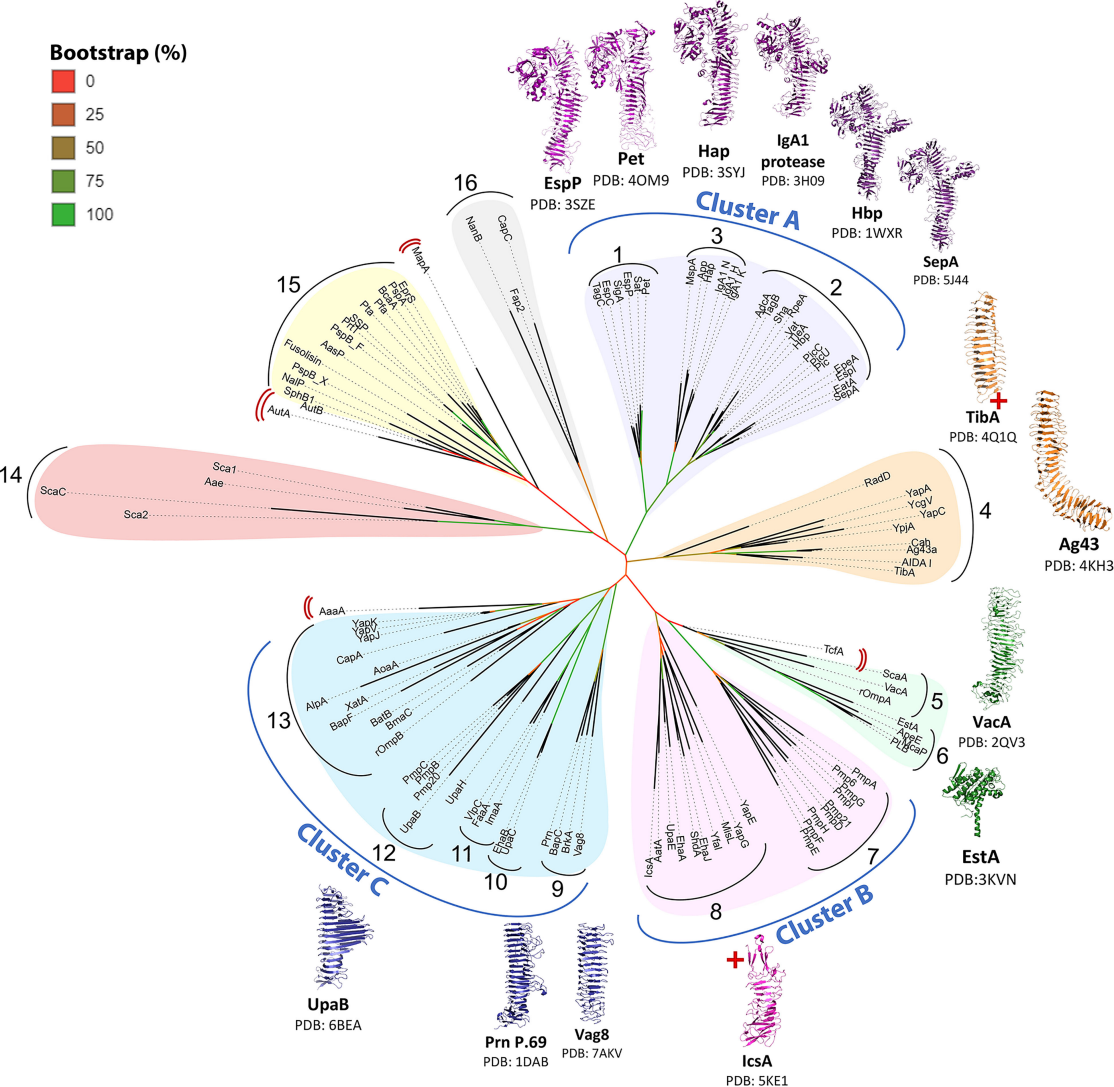
Phylogenetic tree of AT passengers. Unrooted maximum-likelihood phylogenetic tree using Clustal Omega MSA and PhyML with 100 bootstrap iterations and visualized using the interactive tree of life (iTOL). Branch color (red to green) indicates branch support values of 0–90%. Phylogenetic groups are numbered 1─16 with major functional categories indicated by colored shading. 14 published passenger structures are mapped onto the consensus tree, highlighting gaps in structural knowledge. AT structures (54, 70–77, 79–81, 106, 107) were visualized with PyMOL Molecular Graphics System (Schrödinger, LLC) (108). Red cross (**+**) indicates incomplete passenger structure. Red double brackets indicate ungrouped ATs.

To obtain a phylogenetic classification that reflects AT function, following sequence alignment of the 112 curated passengers, an unrooted consensus tree was generated using PhyML (104) with 100 bootstrap iterations and visualized using the interactive tree of life (iTOL) (105). The consensus PhyML tree found the 112 AT passengers formed 16 homologous groupings (**Figure 2**) with 15 of these corresponding to the high-identity groups seen in the multiple sequence alignment pairwise identity heatmap (**Supplementary Figure S2**). The rationale for grouping ATs together took into consideration strong phylogenetic relationships on the tree (cladding together, short branch lengths, and strong bootstrapping support values) as well as similar reported functions and structural features. More distant similarities between nearby groups that share functional themes are considered together as larger clusters. The 16 phylogenetic groups are organized into broad AT functional themes, and importantly show that previously established functional groups form distinct clades: SPATEs (Group 1-2), SAATs (Group 4), GDSL-lipases (Group 6), and subtilisin-like ATs (Group 15). Furthermore, several of these individual clades form part of larger functionally related clusters (Clusters A-C).

Successful identification of these established groups validates the ability of this phylogenetics strategy to distinguish AT groups that share functional and structural similarities. This in turn supports the interpretation of novel groups identified here as functionally related AT classes. The groupings are discussed below, with overall functional themes assigned to each group. **Table S1** provides a comprehensive list of the ATs and their experimentally defined functions.

#### 2.2.1 Cluster A (Groups 1–3): Chymotrypsin-Like Serine Proteases

**Figure 3:**
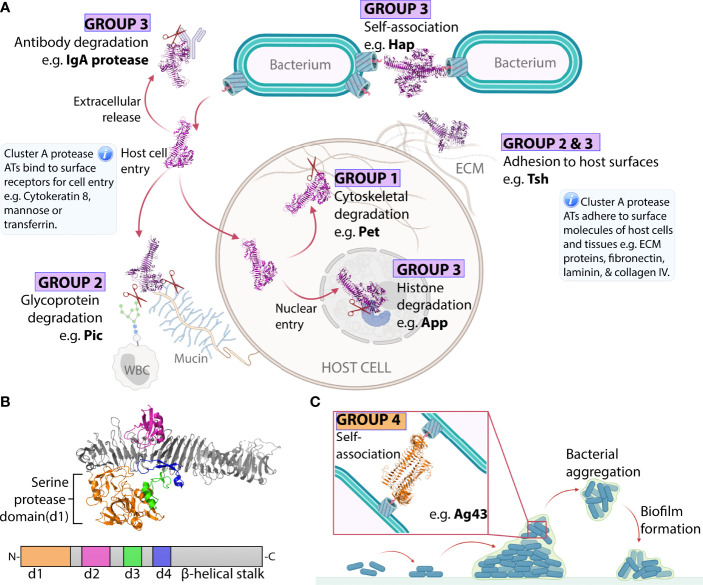
Virulence functions of ATs from Groups 1-4. **(A)** Cluster A chymotrypsin-like protease AT mechanisms. Cluster A protease ATs (Groups 1–3) are released into the extracellular space and move away from the bacterial surface to degrade host proteins. Group 1 proteases then enter host cells and degrade intracellular cytoskeletal components, triggering cytotoxicity. Group 2 proteases remain in the extracellular space where they degrade large host glycoproteins. Group 3 proteases degrade extracellular immunoglobulins or enter host nuclei to degrade nuclear proteins, triggering cell death. Some Cluster A proteases can execute additional functions if they remain at the bacterial surface where they contribute to adhesion to host and bacterial molecules. This includes some members of Group 2 and Group 3, which can promote bacteria-bacteria or bacteria-host adhesion interactions. **(B)** Subdomain organization of a representative Cluster A protease AT. Structure of the Hbp (Group 2) passenger showing the structural elements that are conserved across Cluster A proteases including the β-helical stalk (grey) which acts as a scaffold supporting the globular d1 protease subdomain (orange), the d2 subdomain which resembles a chitin-binding domain (pink), the α-helical loop of the d3 subdomain (green), and the β-hairpin loop of the d4 subdomain (blue). These subdomains are highly conserved, except d2, which is absent from Group 1 proteases. **(C)** Group 4 Self-associating ATs (SAATs) adhesion mechanism. The SAAT Ag43 on adjacent bacterial surfaces self-associate in a molecular Velcro-like manner. This bacteria-bacteria contact contributes to aggregation and biofilm formation. The structures of Hbp (PDB: 1WXR) (75) and Ag43 (PDB: 4KH3) (71) were visualized with PyMOL Molecular Graphics System (Schrödinger, LLC) (108).

Cluster A contains Groups 1–3 totaling 26 ATs belonging to the chymotrypsin-like serine protease family (95). This includes Class-1 SPATEs (Group 1) and Class-2 SPATEs (Group 2) as defined by Ruiz–Perez and Nataro (87). These are now brought together with SPATE-like ATs (SLATs) from outside of the Enterobacteriaceae (Group 3). This is the first time to our knowledge that the close relationship between the SPATEs and SLATs has been shown. This relationship can be interpreted with confidence considering the high branch support values connecting Groups 1–3 (88–95%) and the conservation of well-defined structures among all Cluster A proteases. These are probably the best characterized ATs including six passenger structures (Pet, EspP, IgA1, Hap, SepA, and Hbp) exhibiting similar Type 2 architecture (**Figure 1B**) with a β-helix supporting an N-terminal serine protease subdomain (d1) (72–75, 77, 81). Extended loops arising from the β-helical stalk give rise to further smaller subdomains d2–d4 where d2 resembles a chitin-binding domain, d3 forms an α-helix, and d4 forms a β-hairpin (**Figure 3B**) (87). Recent work revealed that subdomain d3 mediates host cell internalization of Pet from Group 1 by binding cytokeratin-8 to initiate receptor-mediated endocytosis, an essential step in Pet-mediated virulence (109). Currently, no functions have been associated with d2 and d4 subdomains. The finding that the β-helix extended loop that forms d3 is involved in cell binding interactions is consistent with research on the AT adhesins, where their β-helices directly participate in binding interactions (70, 71, 106).

While their clustering together reflects structural conservation, the division of Cluster A proteases into Groups 1–3 reflects their differences.

Group 1 contains six ATs (SigA, EspP, EspC, Pet, Sat, TagC) and encompasses the Class-1 SPATEs described by Ruiz–Perez and Nataro (87). These ATs enter host cells and degrade a vast range of large intracellular host proteins, including cytoskeletal components, which causes cytotoxicity and tissue damage at the site of infection (**Figure 3A**) (110–115). Most originate from diarrheagenic pathogens of the Enterobacteriaceae family where cytotoxicity contributes to cell exfoliation that is characteristic of diarrheal disease. This includes SigA from *Shigella flexneri* (112) alongside EspP, EspC, and Pet from enterohemorrhagic *E. coli* (EHEC), enteropathogenic *E. coli* (EPEC), and enteroaggregative *E. coli* (EAEC) strains, respectively (115–117). Meanwhile, Sat and TagC are expressed by *E. coli* strains associated with urinary tract infections (Sat is also expressed in other pathogens such as enteroaggregative *E. coli* (EAEC) and *Shigella flexneri*) (114, 118).

Group 2 contains 14 ATs (TagB, AdcA, RpeA, Sha, Vat, Hbp/Tsh, TleA, PicC, Pic, PicU, EspI, EpeA, SepA, EatA) and encompasses the Class-2 SPATEs described by Ruiz–Perez and Nataro (87). These ATs primarily cleave extracellular targets including mucin and immune glycoproteins (**Figure 3A**) (91, 119–123). Most originate from enteric pathogens responsible for intestinal infections where mucin degradation increases penetration into the protective mucous layer covering intestinal tissue. This includes PicC and AdcA from *Citrobacter rodentium* (119, 124), SepA from *Shigella flexneri* (125), alongside ATs from *E. coli* strains including EpeA from EHEC (122), TleA and EatA from enterotoxigenic *E. coli* (ETEC) (120, 126), EspI from Shiga toxin-producing *E. coli* (STEC) (127), Pic from *Shigella flexneri* and EAEC (128), and RpeA from rabbit-specific EPEC (REPEC) (129). Meanwhile, ATs such as Sha, TagB, PicC, Hbp, and Vat derive from extraintestinal pathogenic *E. coli* strains (114, 124, 130, 131), that cause urinary tract infections and wound formation (132). Hbp (haemoglobin protease), first found in a human *E. coli* pathogen (EB1) isolated from a peritoneal would infection, shares 99.8% identity with Tsh (temperature-sensitive hemagglutinin), which originates from the avian pathogenic *E. coli* which causes severe respiratory disease in avian populations (75, 130).

Group 3 contains five ATs and encompasses the SPATE-like ATs (SLATs) (MspA, Hap, App, IgA1 proteases). SLATs have properties found in both Class-1 and Class-2 SPATEs (**Figure 3A**). These ATs are expressed by pathogens that infect mucosal epithelia and may become invasive to cause severe disease. For example, App and MspA derive from *Neisseria meningitidis*, while IgA protease and Hap derive from *Haemophilus influenzae* (133–135). These are respiratory pathogens that can disseminate to cause meningitis (136–138). IgA protease is also expressed by *Neisseria gonorrhoeae*, a urogenital pathogen that can spread to cause septic arthritis and endocarditis (139, 140). SLAT functions are well-suited to such pathogens including immune evasion and adhesion to host and bacterial surfaces, which promotes mucosal colonization, as well as tissue damage, which is often required for dissemination.

Specifically, Hap has been shown to adhere to host surfaces and increase aggregation, while App and MspA bind to and enter host cells, degrade histone proteins in the nucleus, and trigger cell death which likely causes tissue damage (81, 141–145). Meanwhile, the IgA1 proteases degrade IgA, which is the most abundant immunoglobulin and an important line of defense at mucosal surfaces (141, 146, 147).

#### 2.2.2 Group 4: Biofilm Forming AT Adhesins

Perhaps the most striking feature of AT adhesins is their sequence diversity despite overall conservation of Type 1 β-helical passenger architecture (**Figure 1B**) in all published structures (**Figure 2**) (70, 71, 76, 79, 80, 106). This diversity underlies their dispersal into 11 phylogenetic groups. Of these, the best studied adhesins are the SAATs encompassed by Group 4. SAATs Ag43, Cah, TibA, and AIDA-I are expressed by *E. coli* where they self-associate with other SAATs on adjacent bacterial surfaces to promote aggregation and biofilm formation (**Figure 3C**) (65, 89, 148–150). These prototypical SAATs are close together within Group 4, which reflects their functional and structural similarities (71, 80, 150–153). Group 4 includes four additional ATs YapC, YpjA, YcgV, YapA, and RadD, all of which are associated with biofilm formation except YapA for which no biofilm studies have been published (154–158). These proteins may be novel members of the SAAT class given their proximity to prototypical SAATs and functional role in biofilm formation. However, the mechanism used to promote biofilm formation remains unknown and structural studies have not been published for YpjA, YcgV, YapA, or RadD. Using PSIPRED (101) we predict a β-helix structure along the full length of the passenger for each of these proteins, which is consistent with the Type 1 AT structure observed in SAATs.

Most Group 4 ATs derive from pathogenic *E. coli* including diarrheagenic strains. This includes YpjA from EHEC (155), TibA from ETEC (159), and AIDA-I from EPEC (160). Meanwhile, Ag43 is one of the most prevalent AT adhesins across many *E. coli* subtypes (21) and YcgV was first identified in the *E. coli* K-12 laboratory strain (156). Conversely, YapC and YapA are expressed by *Yersinia pestis*, the causative agent of pneumonic, septicemic, and bubonic plague (154, 157). Finally, RadD is the only member of Group 4 originating outside the Proteobacteria phylum, being expressed by *Fusobacterium nucleatum*, which contributes to periodontal disease (158). Notably, the SAAT mechanism has only been characterized for ATs from *E. coli* (71, 161, 162). Future studies should determine if YapC, YapA, and RadD use an Ag43-like dimerization mechanism to expand our understanding of ATs adhesins in important pathogens other than *E. coli* (70, 76, 106).

Ag43 is possibly one of the best studied AT in Group 4 and the AT family more broadly. A high-resolution structure of the Ag43a passenger from uropathogenic *E. coli* revealed an L-shaped β-helix forming head-to-tail homodimers through ‘Velcro-like’ non-covalent interactions along the β-helix (71). Ag43 homologues from other *E. coli* pathogens are now known to follow a similar mechanism of interaction to that of Ag43a (161, 162). It is expected that similar modes of action exist for the other ATs in this group such as TibA and AIDA-1 (89). Apart from self-interactions, some of the ATs in this group can also promote binding to host surfaces (152, 153, 159). How the self-interaction binding is coordinated with binding to host surfaces is unknown. Nevertheless, the Ag43a self-interaction mechanism was one of the first clear indications that the β-helix can directly participate in AT function, and since this time AT β-helices from other groups have been shown to participate in diverse binding interactions (70, 106).

#### 2.2.3 Group 5 VacA and Homologs

**Figure 4:**
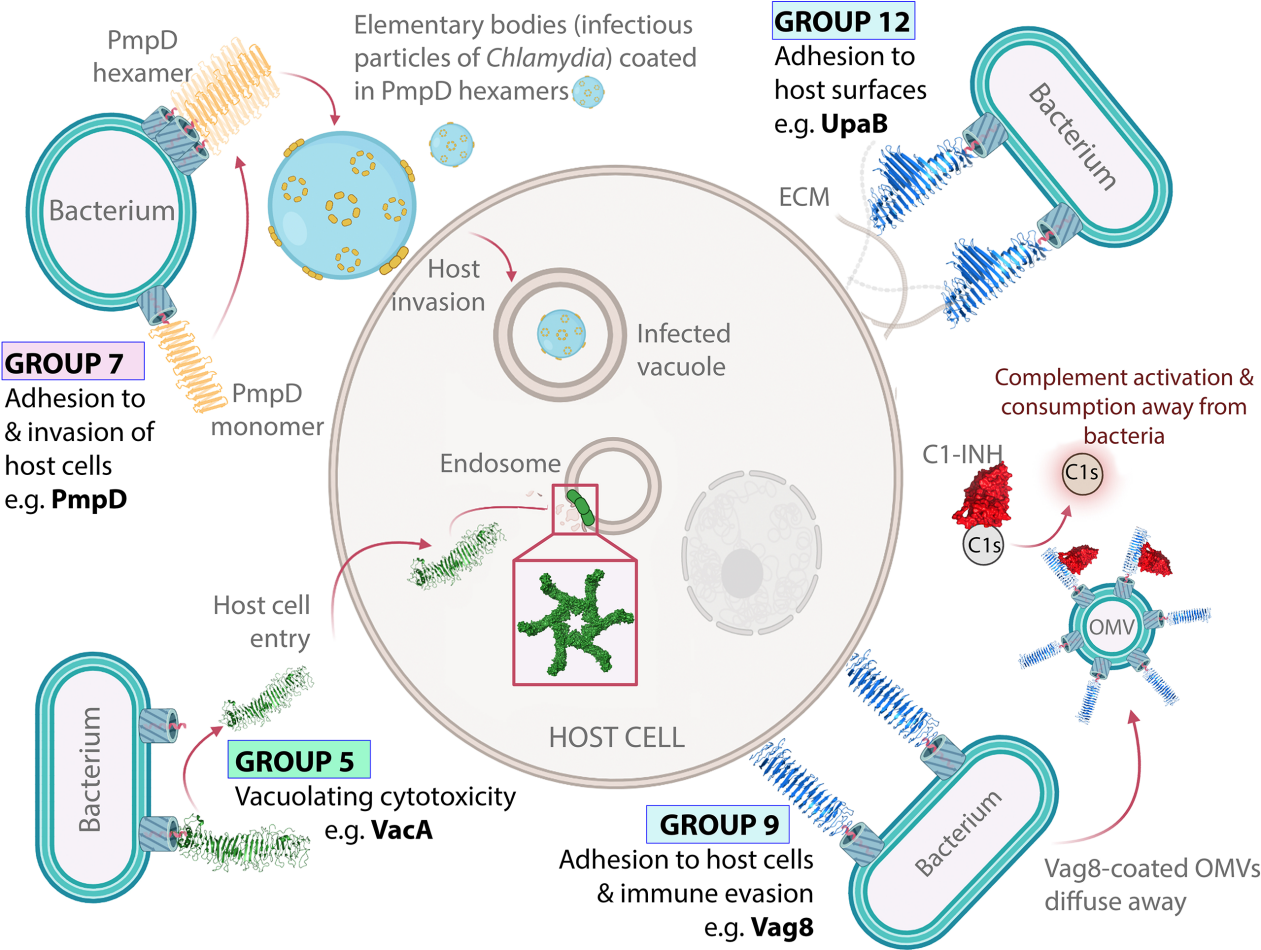
Virulence functions of ATs from Groups 5–12. VacA forms oligomeric pores in intracellular host membranes. VacA (Group 5) forms oligomeric pores in host intracellular membranes including endosomes through horizontal interactions in the lipid bilayer. PmpD is an oligomeric host adhesin. PmpD (Group 7) forms oligomeric rings within the bacterial OM and facilitates host cell invasion. Oligomeric ring structures based on electron microscopy images published by Swanson, et al. (168). Vag8 displays dual immunomodulation and adhesion activities. Vag8 (Group 9) binds to and inhibits the host immune regulator C1-inhibitor (C1-INH), which perturbs the host immune response. Vag8 also promotes adhesion to host cells through an unknown binding interaction. UpaB allows uropathogenic *E. coli* to bind directly to the urogenital epithelia. UpaB (Group 12) binds to ECM proteins on the surface of epithelial cells, which allows bacteria to bind directly to host surfaces within the urogenital tract, thus promoting disease (70). The structures of VacA (PDB: 6NYF) (107), Vag8 (PDB: 7AKV) (106), and UpaB (PDB: 7AKV) (70) were visualized with PyMOL Molecular Graphics System (Schrödinger, LLC) (108).

The best characterized protein in Group 5 is VacA, owing to its important role as a pore-forming toxin during *Helicobacter pylori* gastric infection (163–165). The VacA mechanism of action has been reviewed extensively elsewhere (166). Briefly, after being released from the OM, VacA enters host cells to form oligomeric pores in intracellular host membranes, thereby causing vacuolating cytotoxicity (166). A crystal structure of a VacA fragment (residues 388–844), revealed a β-helical passenger structure (78). This was validated by a cryo-EM structure of full-length VacA, which showed that the remainder of the passenger continued into a right-handed β-helix. Importantly, cryo-EM showed that the VacA membrane pore is formed by homo-hexameric rings through interactions between the N-terminal region of each β-helix, with this region also responsible for making contact with the host membrane (107, 167) (**Figure 4**). Other Group 5 ATs include, ScaA from *Orientia tsutsugamushi*, which causes scrub typhus, and rOmpA from *Rickettsia rickettsii*, which causes rocky mountain spotted fever (169, 170). Although less is known about these proteins, both mediate adhesion to host epithelial cells (169–171) and PSIPRED (101) predictions indicate β-helix structure along both passengers, suggesting structural similarity to the β-helical VacA.

#### 2.2.4 Group 6 GDSL-Lipases

Group 6 encompasses the GDSL-lipases EstA, ApeE, PLB, and McaP, all of which exhibit esterase activity catalyzing the hydrolysis of generic lipid substrates (172–175). Although their biological substrates remain unknown, Group 6 ATs may have a broad role in damaging the phospholipids of host cell membranes (90). Given their small size (<300 aa) and that they largely remain tethered to the outer membrane, the activities of these lipases are likely restricted to the immediate bacterial surface (172–176). The lipolytic activity of EstA has been associated with lipid biosynthesis, bacterial motility, and biofilm regulation (172). Meanwhile, McaP in addition to lipolytic activity also promotes bacterial adhesion to host cells (175, 176). The EstA crystal structure revealed the first non-β-helical AT passenger, whereby the protein is predominantly α-helical due to the GDSL-lipase domain which is directly connected to the α-helical AT linker domain (54). Among published structures, EstA is the only example of Type 3 passenger architecture comprising a catalytic domain without a β-helical stalk (**Figure 1B**). InterPro (102) predicted the lipase domain occupies the entire length of the passenger for ApeE, PLB, and McaP while PSIPRED (101) did not predict β-solenoid structure in this region, suggesting a non-β-helix structure similar to that of EstA. Although this is the only structural evidence of classical ATs lacking a β-helix, this is not uncommon in the wider T5SS. However, outside of the Va group, α-helical ATs tend to form much larger overall structures (17). All Group 6 ATs derive from γ-proteobacteria including EstA from *Pseudomonas aeruginosa*, an opportunistic pathogen associated with nosocomial infections (172), ApeE from *Salmonella enterica* Typhimurium, which causes the diarrheal disease salmonellosis (173), PLB from *Moraxella bovis*, which causes infectious bovine conjunctivitis (174), and McaP from *Moraxella catarrhalis*, which causes otitis media and upper respiratory tract infections (175, 176).

Notably, the clades for Groups 5 and 6 are close together, linked with strong branch supports in the phylogenetic tree (**Figure 2**) and can share up to 20% local amino acid identity. However, they are not known to share structural or functional similarities. The proximity of these distinct groups is therefore striking, and their sequence similarities are not confined to local regions or motifs, but rather spread throughout the sequences, possibly inferring a distant evolutionary relationship (data not shown).

Not shown within the tree but included within this group is the GDSL-lipase BatA from *Burkholderia* (177). BatA with only up to 28% sequence identity to members of this group, positions at its margins. Notably, BatA also shares significant sequence identity to the Group 13 adhesins.

#### 2.2.5 Cluster B (Groups 7–8): Adhesins

Cluster B encompasses Groups 7 and 8 containing ATs that function as adhesins. Host binding is common to all Cluster B ATs while many Group 8 ATs also contribute to bacterial aggregation and/or biofilm formation (155, 156, 178–195). Furthermore, PSIPRED (101) predicted β-helix structure for all Cluster B passengers, which is consistent with the β-helical structure observed in the partial structure of IcsA (79).

Group 7 contains nine ATs designated ‘polymorphic membrane proteins’ (Pmps) including Pmp6 and Pmp21 from *Chlamydia pneumoniae* along with PmpA, PmpD, PmpE, PmpF, PmpG, PmpH, and PmpI from *Chlamydia trachomatis.* These are typically OM-anchored ATs that promote host cell adhesion and invasion, consistent with the intracellular lifestyle of the *Chlamydia* spp. from which they are derived (178, 179, 181, 196). Beyond this broad function, most Pmps are poorly characterized with no published structures. However, PmpD and Pmp21 have been observed to form higher-order oligomers (168, 197, 198). For PmpD, these oligomers appear as flower-like rings in the bacterial OM (168) (**Figure 4**). Notably, VacA, which is placed nearby in Group 5, is also known to form flower-like oligomers within lipid bilayers (199). This oligomerization may be important in the Pmp binding mechanism, however, the functional significance of PmpD and Pmp21 oligomerization has not been well established. Pmp21 is the only Group 7 AT where the binding partner required for host cell entry is known as it has been shown to promote invasion of host cells by binding to epidermal growth factor receptor (EGFR) (180).

Group 8 consists of ten proteins, YapE, MisL, YapG, Yfal, ShdA, EhaJ, UpaE, EhaA, IcsA and AatA, most of which derive from Enterobacteriaceae that cause diarrheal disease. This includes EhaA and EhaJ from diarrheagenic *E. coli* (155, 194), ShdA and MisL from *Salmonella enterica* Typhimurium (184, 193), and IcsA from *Shigella flexneri* (200). Others including AatA, YfaL, and UpaE derive from extraintestinal *E. coli* (156, 183, 195). Group 8 ATs that are found outside the Enterobacteriaceae family, include YapE from *Yersinia pestis* and YapG from *Yersinia pseudotuberculosis*, the latter causing Far East scarlet-like fever (157, 188).

Group 8 proteins are outer membrane anchored and primarily act as adhesins, with many having dual binding abilities to both host and bacterial targets. Specifically, most, including YapE, MisL, ShdA, EhaJ, UpaE, EhaA, IcsA and AatA mediate host adhesion (155, 183, 184, 186–189, 191–195, 201). For ShdA, MisL, EhaJ, and UpaE, this involves binding to extracellular matrix (ECM) proteins (184, 186, 187, 193–195). Whether ECM binding is a common host binding mechanism across Group 8 remains unknown as binding partners on host epithelial surfaces have not been published for YapE, EhaA, IcsA, and AatA. However, a host intracellular target of IcsA is known, Neural Wiskott–Aldrich syndrome protein (N-WASP), which contributes to the regulation of actin polymerisation as part of the cell cytoskeleton (202). IcsA activates N-WASP to promote intracellular actin-based spread of *S. flexneri* through the colonic epithelial layer. Regarding bacterial aggregation and/or biofilm formation, all but ShdA are associated with this phenotype (155, 156, 182, 188, 190, 192, 194, 195). However, the mechanism by which these ATs promote bacterial aggregation/biofilm formation has not been determined. IcsA promotes both biofilm formation and forms homodimers, which has raised the possibility of self-association similar to that of Ag43a (190, 203). However, a link between IcsA dimerisation and biofilm formation has not been established and dimerisation has not been demonstrated for other group members. Furthermore, the only passenger structure for Group 8 is a small IcsA fragment (residues 419–758) in the monomeric form, providing no insight into self-association (79).

#### 2.2.6 Cluster C (Groups 9–13): Adhesins

Cluster C (Groups 9–13) contain a separate cluster of adhesin ATs that are primarily anchored to the outer membrane where their predominant function is adhesion to host cells and/or surfaces. Currently, Groups 10, 11, and 13 lack published structures.

Group 9 contains four ATs (Vag8, BrkA, Prn, and BapC), all of which derive from *Bordetella* spp. and exhibit high conservation in sequence, structure, and function. The reported crystal structure of Prn (76) and the cryo-EM structure of Vag8 (106) both reveal Type 1 AT β-helices. Meanwhile, PSIPRED (101) predicts β-helical passengers for BrkA and BapC, which is also consistent with Type 1 AT β-helices.

Group 9 ATs exhibit dual host adhesion and immune evasion activities (69, 204–206). For Prn, host binding involves its RGD integrin-binding motif (205). BrkA, BapC, and Vag8 also contain RGD motifs, suggesting a possible common host binding mechanism (206–208). To date, the host factors recognized by Group 9 ATs to promote cell adhesion are unknown. Furthermore, while evasion of the innate immune response is also common among Group 9 ATs, each is unique in its approach. Prn affords protection from the inflammatory response and neutrophil-mediated clearance (209, 210). Meanwhile, BapC, Vag8, and BrkA promote serum resistance by reducing complement-mediated killing (68, 208, 211, 212). The Vag8 immune evasion mechanism is the best understood. Vag8 enhances serum resistance by inhibiting the serpin C1-inhibitor (C1-INH) (106, 212), which regulates the complement system (68, 212). Structural studies have shown that Vag8 binds C1-INH using extended loops lining one face of its β-helix (106), thus providing further evidence that β-helix structures can directly participate in AT functions.

Although Group 9 ATs are present at the outer membrane, growing evidence suggests *Bordetella* may deploy ATs (i.e., Prn, BrkA, and Vag8) in OMVs, disseminating AT function away from the bacterial surface (68, 213, 214). This finding has been crucial for understanding Vag8 function. Hovingh, et al. (68) proposed that OMVs coated with Vag8 block C1-INH and enable unregulated complement activation away from the bacterial surface, thus protecting bacteria by depleting complement factors before they can be deposited on the bacterial surface (**Figure 4**).

Group 10 contains two ATs derived from pathogenic *E. coli*, UpaC and EhaB, both of which promote biofilm formation (215, 216). In addition, EhaB also mediates host adhesion by binding to ECM proteins (155). Group 11 contains three ATs (FaaA, VlpC, ImaA) that increase murine gastric colonization by *H. pylori* (217). Their placement in Cluster C suggests their contribution to colonization may involve host adhesion, aggregation, or biofilm formation. Unfortunately, to date, little is known about the mechanism of action of Group 10 and 11 ATs.

Group 12 comprises five ATs that promote host adhesion, UpaB, UpaH, PmpB, PmpC and Pmp20 (178, 179, 215, 218, 219). For UpaB and UpaH, both of which derive from uropathogenic *E. coli*, this involves binding to host ECM proteins (215, 218, 219). Meanwhile, the less-defined members PmpB, PmpC, and Pmp20 promote adhesion and entry of Chlamydia into host cells (178, 179). However, ECM binding or biofilm formation studies have not been conducted for the Pmps. The best-studied member of Group 12 is UpaB, which promotes bladder colonization through direct adhesion to urogenital epithelia (215). The crystal structure of the UpaB passenger is consistent with a Type 1 AT β-helix (70). However, its structure reveals unique features, in particular long loops and β-strand extensions projecting out from the β-helix, which form a long hydrophilic groove (70). UpaB was found to bind polysaccharides at this site, and in silico modelling and the resemblance of this groove to the active site of glycosaminoglycan (GAG) lyases, suggests that UpaB binds GAGs lining the human uroepithelium using this binding groove (70). In addition, on the opposite side of UpaB’s β-helix is a second binding site which was shown to bind human fibronectin. Altogether, this demonstrates that residues within the UpaB β-helix contribute to two host binding sites that promote urinary tract colonization. UpaB is therefore an excellent example of an AT β-helix exhibiting multiple direct contributions to the virulence phenotype.

Group 13 contains 11 ATs (CapA, YapJ, YapK, YapV, rOmpB, BatB, BmaC, XatA, BapF, AoaA, AlpA), most of which are anchored to the bacterial surface and function as adhesins. Notably, this is the largest adhesin group in the present study and the most diverse in sequence identity (ranging from 0–81%), passenger length (ranging from 280–3333 aa), and taxonomically with ATs deriving from ten Genera: *Yersinia*, *Campylobacter*, *Pseudomonas*, *Brucella*, *Bordetella*, *Rickettsia*, *Helicobacter*, *Azorhizobium*, *Burkholderia*, and *Xylella* (83, 177, 220–227). This covers a wide range of bacteria, from *H. pylori*, among the most widespread and oldest human pathogens and a major cause of stomach cancer worldwide (227), to *Xylella fastidiosa*, a genus of plant pathogens that is rapidly spreading across the globe and destroying important agricultural crops with huge economic impacts (225). This diversity is reflected by the bootstrapping values with Group 13 showing the lowest within-group bootstrapping among the Cluster C adhesins (**Figure 2**).

Consistent with the rest of Cluster C, PSIPRED (101) predictions indicate β-helical passenger structure for the majority of Group 13 ATs. However, notable exceptions include AlpA which has been predicted to be α-helical. Another unusual feature only shared by AlpA and CapA in this group includes the lipidation at the N-terminus of their mature passengers (220, 227). Lipidation is thought to allow the passengers to remain associated with the bacterial surface (98), a characteristic which would be favorable for an adhesin.

Overall, the reported functions for Group 13 ATs broadly resemble those of other Cluster C adhesins. Specifically, BapF and XatA promote bacterial aggregation and/or biofilm formation (225, 226). Meanwhile, YapJ, YapK, YapV, CapA, BmaC, rOmpA, and AlpA promote host adhesion, including ECM binding for the Yaps and BmaC (220–222, 224, 226–228). Additionally, BatB binds immunoglobulins to perturb the human immune response (223), while AoaA promotes the symbiotic relationship between legume root nodules and rhizobia by dampening plant defenses (83). While these immunomodulatory activities are somewhat reminiscent of the dual action adhesins and immunomodulators of Group 10, the adhesive properties of BatB and AoaA have not been reported.

Collectively, although Group 13 ATs display related functional properties, these proteins are very diverse and their phylogenetic relationships with well characterized ATs are uncertain, which warrants further studies on this AT grouping.

#### 2.2.7 Group 14: α-Helical Adhesins

Our phylogenetic analysis identified a separate clade containing four surface-bound ATs that contribute to host adhesion including Aae from *Acintobacillus actinomycetemcomitans* (96) alongside Sca1, Sca2, and ScaC from Rickettsiaceae (229–231). Other functions associated with Group 14 include biofilm formation for Aae and intracellular invasion and motility for Sca2 (232, 233). Mechanistically, ATs in this group are poorly characterized and no structures are currently available in the PDB. Interestingly, PSIPRED (101) analysis predicts α-helical passenger structures for all Group 14 ATs, distinguishing this group as a type Va AT subfamily composed only of α-helical adhesins.

#### 2.2.8 Group 15: Subtilisin-Like Serine Proteases

Group 15 contains 13 subtilisin-like protease ATs with remarkably diverse taxonomic backgrounds primarily deriving from β- and γ-proteobacteria. This includes PspB_F, Pfa, BcaA, EprS, and PspA from *Pseudomonas* spp. (234–238), SSP and PrtT from *Serratia marcescens* (239, 240), NalP from *N. meningitidis* (59), SphB1 from *B. pertussis* (241), AasP from *Actinobacillus pleuropneumoniae* (242), PspB_X from *X. fastidiosa* (243), along with Pta from *P. mirabilis* (97). These subtilisin-like ATs are also present in bacteria outside the Proteobacteria phylum as evidenced by the presence of Fusolisin from *Fusobacterium nucleatum* (61). In stark contrast to the Cluster A proteases, the subtilisin-like proteases of Group 15 are among the least understood ATs. Based on secondary structure and conserved domains predicted with PSIPRED (101) and InterPro (102), these ATs are thought to comprise of an ~400 aa N-terminal protease domain followed by an ~200 aa β-helix structure, thus following a Type 2 AT organization similar to the Cluster A proteases. Subtilisin-like ATs are known to have dual roles in bacteria, both at the bacterial surface and when released into the host environment. At the bacterial surface, protease activities of Pfa1, EprS, SphB1, AasP, and NalP are used to process other extra-cytoplasmic proteins including virulence factors (59, 235, 241, 242, 244–246). For example, NalP is responsible for proteolytic maturation of Cluster A protease ATs App, MspA, and IgA1 protease (59, 246). Meanwhile, SphB1 indirectly modifies host adhesion by modifying filamentous hemagglutinin adhesion molecules (241, 245). The capacity of NalP and SphB1 to process these virulence factors, is thought to rely on their abilities to remain temporarily associated with the bacterial surface *via* their lipidation at their N-terminus similar to members of Group 13 (98, 245). After their release from the bacterial surface, subtilisin-like protease activity appears responsible altering host processes. For example, Pta and Pfa promote host cell cytotoxicity (97, 235) and Fusolisin, EprS, PspB_F, Pfa, and NalP contribute to immunomodulation (234, 235, 237, 247, 248). This likely results from degradation of host proteins as Fusolisin degrades IgA whereas NalP cleaves C3 of the complement system (247, 248). Meanwhile, NalP can also enter a range of host cell types where it alters cellular metabolism (249). Notably, cytotoxicity, host cell internalization, and immunomodulation are also features of the Cluster A chymotrypsin-like proteases.

#### 2.2.9 Group 16: Adhesins and a Sialidase

Group 16 contains three bacterial surface associated ATs including CapC from *Campylobacter jejuni* and Fap2 from *Fusobacterium nucleatum*, which promote host adhesion and mediate bacterial aggregation (250, 251). This group also includes NanB from *Pasteurella multocida*, the only AT with defined sialidase activity, thought to benefit in nutrient acquisition (252). PSIPRED (101) analysis predicted β-helix passenger structure for all members, however, this group is poorly characterized in terms of both structure and function. Accordingly, future research may further define the functional classification of the Group 16 ATs. Importantly, unlike all other phylogenetic groups reviewed here, Group 16 did not form a high-identity cluster on the multiple sequence alignment heatmap (**Supplementary Figure S2**). This suggests that Group 16 may be an outgroup of proteins lacking strong homologs in the current pool of functionally investigated ATs.

#### 2.2.10 Ungrouped ATs

Our phylogenetic analysis also uncovered several ATs without strong relationships to any clade, as evidenced by low sequence identity across the AT pool in the sequence alignment heatmap (**Supplementary Figure S2**) and low bootstrap values within the phylogenetic tree (**Figure 2**). For example, the passenger of TcfA, an adhesin from *B. pertussis*, does not share significant identity with any other passenger included in this study. PSIPRED (101) analysis predicted a predominantly unstructured passenger for TcfA, which is consistent with its unusually high proline content (17%). TcfA has been shown to promote *B. pertussis* adhesion to the respiratory tract (69).

The adhesins AutA and AutB share homology with one another but showed no similarity to other AT adhesin groups in either the sequence alignment heatmap (**Supplementary Figure S2**) or the phylogenetic tree (**Figure 2**). These proteins are positioned within the subtilisin-like protease clade (Group 15) but with extremely low branch support values (13%). As such, AutA and AutB remain ungrouped. Functionally, AutA and AutB promote aggregation and biofilm formation in *N. meningitidis* (84, 253, 254). PSIPRED (101) analysis of both AutA and AutB predicts substantial β-helical passenger structure. This is typical of AT adhesins, however their distinction from other adhesins at the sequence level suggests unique structural and functional features.

In addition to the ungrouped adhesins, we found three enzyme classes on the tree with a single AT representative that did not therefore form a large functional group. This includes two enzymes that remain ungrouped: AaaA, a surface-bound arginine-specific aminopeptidase (255), and MapA, an acid phosphatase (256). These enzymes encompass two of the five enzyme classes observed in the phylogenetic analysis with the others being proteases, esterases, and the lone sialidase, NanB (252). NanB is part of Group 16, a probable outgroup of mostly unrelated proteins. Catalytic domain and secondary structure predictions using InterPro (102) and PSIPRED (101), respectively, indicate MapA may adopt a Type 2 AT architecture encompassing an N-terminal catalytic domain with a β-helix C-terminus, while AaaA appears to take on Type 3 AT architecture wherein the catalytic domain spans the full length of the passenger (**Supplementary Figure S3**).

Future structure-function studies on additional proteins in the Type Va AT family may shed some light as to whether these to date unrelated ATs proteins form part of other functional phylogenetic groups yet to be identified.

## 3 Discussion

The T5SS, which involves self-mediated transport of autotransporter (AT) proteins outside the cell, is the simplest system for extracellular secretion in Gram-negative bacteria (13–15). Transport relies on a modular architecture wherein each AT contains a signal peptide, translocator module and a functional passenger. Passenger functions vary widely, conferring functional diversity to this large family of bacterial secreted proteins. Comparatively, translocators are highly conserved where each promotes translocation of a passenger that may possess various structural elements and catalytic domains. This combination of variation and uniformity underlies the robustness of this secretion system: by leveraging both the passenger’s functional flexibility and the translocator’s simple and energetically economical secretion capacity, ATs have evolved into highly specialized molecular tools that promote many aspects of bacterial fitness and pathogenesis.

Steadily increasing numbers of publicly available ATs sequences and publications describing their functional properties prompted us to re-evaluate the classification of this protein family, focusing on their diverse passengers. In this study we show that 112 functionally characterized ATs can be divided into 16 phylogenetic groups. By using the passenger sequences alone, the divisions best reflect common passenger functions, many of which contribute to bacterial virulence. Overall, we found AT enzymes form three main divisions: chymotrypsin-like proteases (Cluster A), subtilisin-like proteases (Group 15), and GDSL-lipase esterases (Group 6). In addition to different enymatic actions, these AT enzymes also exhibited diverse structural compositions. Protease ATs adopt Type 2 passenger structures (**Figure 1B**) wherein an N-terminal protease subdomain responsible for cleaving target proteins sits atop a β-helix for which the functional role is less clear (94). Meanwhile, GDSL-lipases represent Type 3 structure (**Figure 1B**) which includes an esterase domain responsible for hydrolyzing target lipids without any β-helical content (54). Beyond these three main divisions, we observed a further three enzyme classes with a single representative in the pool of characterized ATs, including the aminopeptidase AaaA (ungrouped), the acid phosphatase MapA (ungrouped), and the sialidase NanB (Group 16). Future phylogenetic studies may reveal additional groups that capture these enzyme functions. Most of the remaining ATs are adhesins distributed into 11 groups reflecting a wide range of specialized functions. Based on limited published structural studies, AT adhesins typically exhibit Type 1 structure (**Figure 1B**) with long β-helical passengers (70, 71, 76, 79, 80, 106). Where adhesion mechanisms have been studied at the molecular level, the long β-helix forms an extended binding interface with specific host or bacterial targets, achieving high affinity through the additive effect of many non-covalent interactions (70, 71, 106). In some cases, the β-helix forms a groove along the binding interface to further facilitate specific binding (70, 106). Furthermore, ATs may bind multiple targets using different faces of the β-helix (70). Through these interactions adhesins promote adherence to host surfaces, biofilm formation, or bacterial aggregation. Biofilm formation is most strongly associated with the Group 4 SAATs but is also observed in some Group 8 and Group 10 ATs. Meanwhile, most Cluster B adhesins (Groups 7–8) promote adhesion to host surfaces yet some, including the Group 7 Pmps and IcsA from Group 8, also self-associate to form homo-oligomers. Furthermore, Cluster C adhesins (Groups 9–13) that are not known to oligomerize, include an array of ATs that promote adhesion to host surfaces and less frequently bacterial surfaces. A handful of poorly characterized adhesins are also present in Groups 5 and 16. Meanwhile, Group 14 is predicted to encompass adhesins with α-helical passengers, which has not been described previously for Type Va ATs and requires experimental verification. Importantly, Group 1 and 2 (SPATEs), Group 4 (SAATs), Group 15 (subtilisin-like proteases) and Group 6 (GDSL-lipases) represent previously established classes, which authenticated the phylogeny along with the 11 new groups.

## 4 Conclusion and Future Perspectives

Our work through providing a better understanding into the relationships of AT structure and function has revealed insights into the mechanisms and diversity of ATs, that, importantly, sheds light on the lesser-known ATs. We anticipate that this will aid in the characterization of further ATs and has also identified groups of ATs that require further research attention. This is particularly true of the six functional groups that entirely lack published structures and detailed mechanisms of action (Groups 7, 11, 13, 14, 15, and 16). Following the trend observed for other groups, we would expect these six groups to reveal new types of AT structures and modes of action. Although our pool of 112 sequences only represents a fraction of the >1500 ATs that have already been identified (14), our use of only ATs with some functional characterization performed should increase the reliability of our findings. This in itself also highlights the overall lack of knowledge regarding ATs, with most still uncharacterized especially outside of *E. coli*. Unfortunately, this may have also created some bias in our study and also contributed to the findings such as the lack of characterized homologs for functional outliers such as NanB (sialidase), MapA (acid phosphatase) and AaaA (aminopeptidase), which are likely representatives of separate functional groups. Apart from an increased awareness surrounding ATs, our work has also shed further light on bacterial pathogenesis and could be used to develop new technologies including antimicrobials and vaccines. Currently, the classical AT Prn is used in pertussis vaccines including Boostrix^®^, Infantrix^®^, and Adacel^®^ (257–259), and the trimeric AT NadA is included in the meningococcal vaccine Bexsero^®^ (260). ATs have also been identified as useful targets for anti-virulence antimicrobials (261). However, efforts to target ATs have been perhaps hampered by the scarcity of molecular-level knowledge. This can be observed in the biotechnological applications of ATs, which primarily exploit the relatively well-defined translocation mechanism for secretion or surface display of recombinant proteins such as β-lactamase (262) and DNA polymerase (263) amongst others (264–266). Further, the ATs have been used to engineer live bacteria that secrete a peptide therapeutic (267). The detailed protein structure for Hbp also allowed engineering of the passenger for multivalent antigen display on OMV-based vaccines (268–270). Overall, this work has provided an updated perspective of AT classification, that informs on AT functional relationships, which could benefit antimicrobial and vaccine research, but above all hopefully inspire further research into this area of widespread and abundant bacterial proteins.

## Author Contributions

BH and JP contributed to conception of the study. KC, JP, and BH contributed to the design of the study. KC compiled the database of protein sequences and functions and performed the bioinformatics and phylogenetic analyses. KC wrote the first draft of the manuscript. JP, BH, and LH wrote sections of the manuscript. All authors contributed to manuscript revision, read, and approved the submitted version.

## Funding

This work was supported by the Australian Research Council (ARC) project grants (DP180102987, DP210100673), a National Health and Medical Research Council (NHMRC) Project Grant (GNT1143638) and an La Trobe Strategic Innovation Fund project.

## Conflict of Interest

The authors declare that the research was conducted in the absence of any commercial or financial relationships that could be construed as a potential conflict of interest.

## Publisher’s Note

All claims expressed in this article are solely those of the authors and do not necessarily represent those of their affiliated organizations, or those of the publisher, the editors and the reviewers. Any product that may be evaluated in this article, or claim that may be made by its manufacturer, is not guaranteed or endorsed by the publisher.
