# Supplementary material for: Phylogenetic Classification and Functional Review of Autotransporters
Source: Front Immunol. 2022 Jul 1;13:921272. doi: 10.3389/fimmu.2022.921272 (PMC9289746; doi:10.3389/fimmu.2022.921272)
Supplement: Supplementary file 1 [file Presentation_1.pdf]

Supplementary Data

Phylogenetic Classification and Functional Review of Autotransporters

Kaitlin R. Clarke[1], Lilian Hor[1], Akila Pilapitiya[1], Joen Luirink[2], Jason J. Paxman[1*], Begoña Heras[1*]

[1]Department of Biochemistry and Chemistry, La Trobe Institute for Molecular Science, La Trobe University, Melbourne, VIC, Australia.

[2]Department of Molecular Microbiology, Amsterdam Institute of Molecular and Life Sciences (AIMMS), Vrije Universiteit, De Boelelaan 1085, 1081 HV, Amsterdam, The Netherlands

*** Correspondence:** Begoña Heras, b.heras@latrobe.edu.au; Jason Paxman, j.paxman@latrobe.edu.au

Figures Supplementary 1 to 3

Table Supplementary 1

References

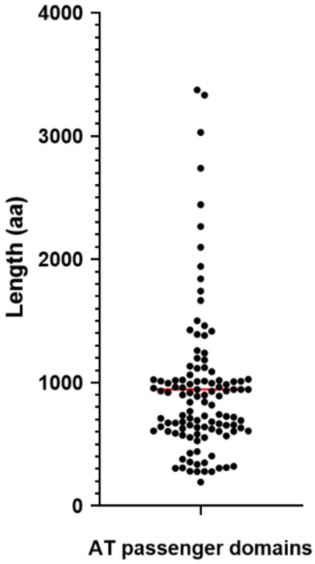

Figure S1. Passenger domain lengths. Scatter plot of passenger domain lengths for 112 analyzed ATs ranging from 193 to 3393 amino acid residues (aa). Red line indicates the mean.

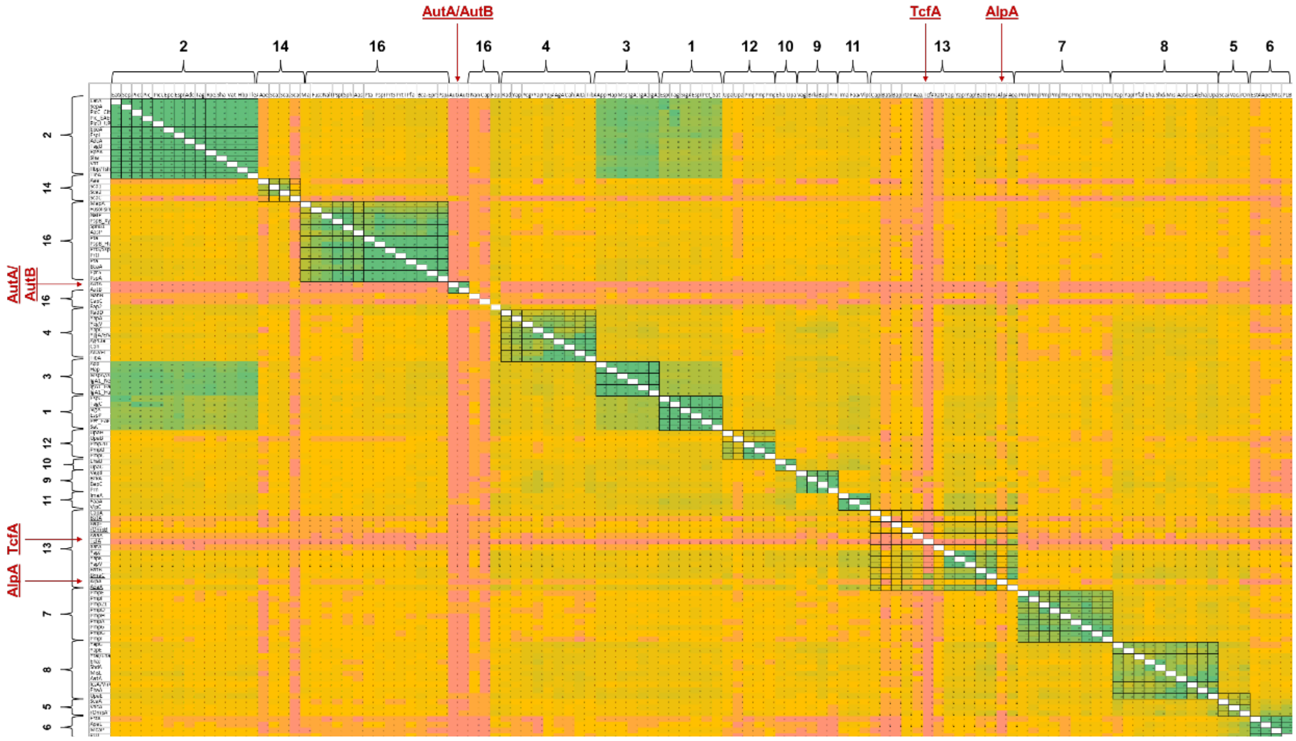

Figure S2. Heatmap of pairwise identities between 112 analyzed AT passenger domains. MSA was generated with Clustal Omega multiple sequence alignment. Coloring indicates identity ranging 0–96% (red–green). High-identity groups form green clusters.

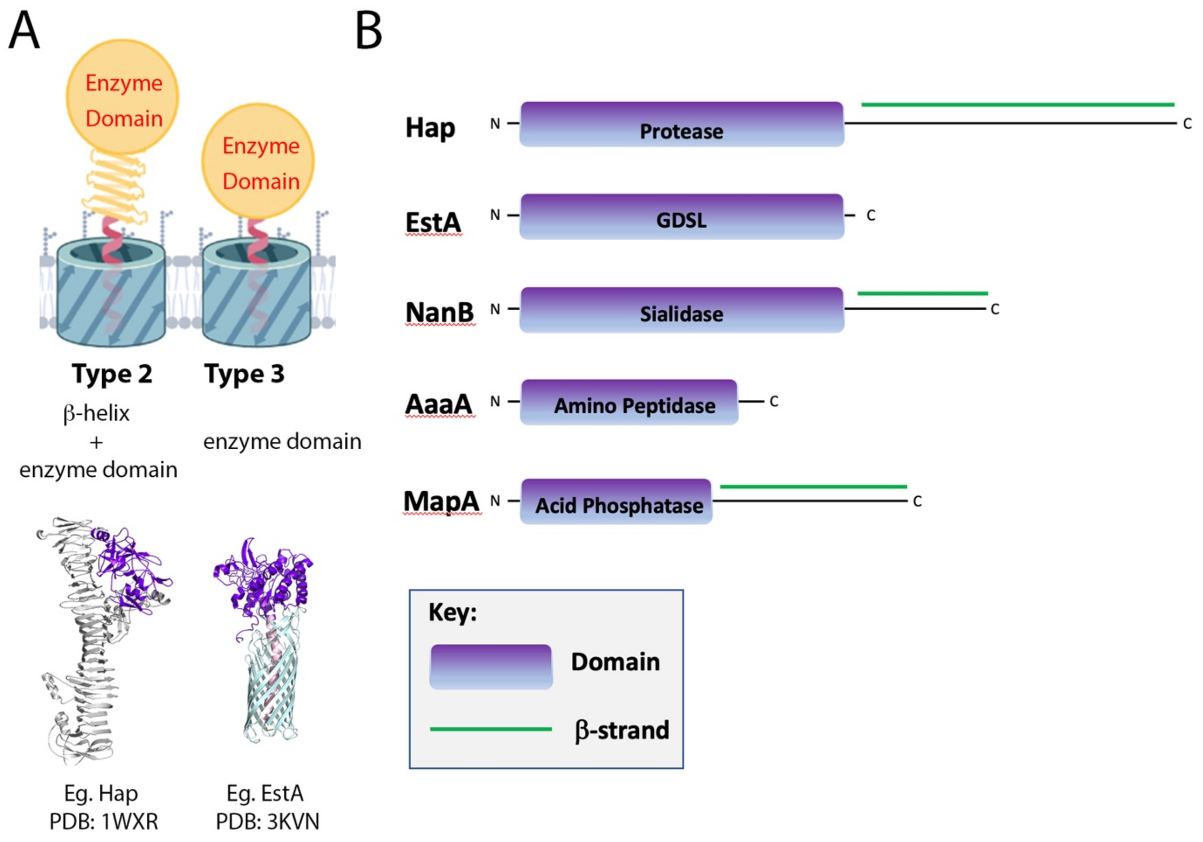

Figure S3. Predicted passenger domain architecture of ungrouped ATs. A. Type 2 and 3 configurations of classical Va AT passengers: Type 2 encompass a catalytic domain at the N-terminus of the β-helix; Type 3 structures lack a β-helix and the catalytic domain is connected to the translocator via the linker helix. **B.** Catalytic domain and secondary structure predictions of ungrouped ATs using InterPro(1) and PSIPRED(2), respectively, indicate **MapA** and **NanB** may adopt a Type 2 AT architecture encompassing an N-terminal catalytic domain with a β-helix C-terminus. **AaaA** appears to adopt a Type 3 AT architecture as the catalytic domain spans the full length of the passenger domain.

Supplementary Data Function and classification of Autotransporters

Table S1

Name	G[a]	Reported function	*Species*	SS[b]	ID[c]
**TagC**	**1**	- Serine protease, cytotoxicity involving alteration of the actin cytoskeleton (3, 4)	*Escherichia coli* (ExPEC)	αβ	QBB20996.2
**SigA**	**1**	- Serine protease, cytotoxicity (5) via degrading intracellular host fodrin, which alters the actin cytoskeleton (6)	*Shigella flexneri*	αβ	AAF67320.1
**EspC**	**1**	- Serine protease, cytotoxicity via degrading intracellular host fodrin, which alters the actin cytoskeleton (7, 8)	*Escherichia coli* (EPEC)	αβ	AAC44731.1
**Pet**	**1**	- Serine protease, cytotoxicity via degrading intracellular host fodrin, which alters the actin cytoskeleton (9, 10)	*Escherichia coli* (EAEC)	αβ	CBG27789.1
**EspP (PssA)**	**1**	- Serine protease, cytotoxicity (11)<br>- Pro-hemorrhagic and anti-coagulation activity (12-14)	*Escherichia coli subtype EHEC*	αβ	CAA66144.1
**Sat**	**1**	- Serine protease, vacuolating cytotoxicity (15, 16) via degrading intracellular host fodrin (17), which alters the actin cytoskeleton (18)	*Escherichia coli subtype UPEC*	αβ	AAG30168.1
**RpeA**	**2**	- Serine protease (19)<br>- Adhesion to host epithelial cells (19)	*Escherichia coli subtype REPEC*	αβ	AAT36311.1
**Pic**	**2**	- Serine protease, degradation of mucin (20) and immune glycoproteins (21)<br>- Serum resistance via degradation of complement proteins C3, C4, and C2 (22)<br>- Hemagglutination (20)	*Escherichia coli subtype EAEC*	αβ	ALT57188.1
**PicU**	**2**	- Serine protease, degradation of mucin (23)	*Escherichia coli* (UPEC)	αβ	AAN78833.1
**PicC**	**2**	- Serine protease, degradation of mucin and immune glycoproteins (21)<br>- Immune evasion via TLR2 inhibition (24)	*Citrobacter rodentium*	αβ	WP_01290893 3.1
**Hbp (Tsh)**	**2**	- Serine protease, degradation of mucin (25), immune glycoproteins (21) and haemoglobin (26)<br>- Hemagglutination (27)<br>- Adhesion to host epithelial cells (3) and ECM (28)<br>- Biofilm formation (3)	*Escherichia coli* (EB1)	αβ	CAA11507.1
**Sha**	**2**	- Serine protease, mucin degradation, cytotoxicity, adhesion to and entry into host epithelial cells, hemagglutination, biofilm formation, bacterial aggregation (3, 4)	*Escherichia coli O1:K1 strain QT597 (ExPEC)*	αβ	QDB64255.2
**TagB**	**2**	- Serine protease, cytotoxicity, adhesion to and entry into host epithelial cells, hemagglutination, bacterial aggregation (3, 4)	*Escherichia coli O1:K1 strain QT598 (ExPEC)*	αβ	QBB20986.2
**AdcA**	**2**	- Serine protease, immune glycoprotein degradation (21)<br>- Adhesion to host epithelial cells (29)<br>- Bacterial aggregation (29)	*Citrobacter rodentium*	αβ	CBG90828.1
**TleA**	**2**	- Serine protease, degradation of mucin and immune glycoproteins, adhesion to host epithelial cells (30)	*Escherichia coli subtype ETEC*	αβ	AGX26722.2
**EatA**	**2**	- Serine protease, highly specific degradation of MUC2 mucin (31) and EtpA, a glycoprotein adhesin and ETEC virulence factor (32)	*Escherichia coli subtype ETEC*	αβ	CAI79539.1
**EpeA**	**2**	- Serine protease, mucin degradation (33)	*Escherichia coli subtype EHEC*	αβ	AAL18821.1
**SepA**	**2**	- Serine protease, promotion of intracellular invasion via disrupting host epithelial cell-cell contacts (34)	*Shigella flexneri*	αβ	ADA76801.1
**EspI**	**2**	- Serine protease, pepsin and apolipoprotein degradation (35)	*Escherichia coli subtype STEC*	αβ	CAC39286.1

Supplementary Data Function and classification of Autotransporters

**Vat**	**2**	- Serine protease, degradation of mucin (21) and immune glycoproteins (21)<br>- Intracellular vacuolating cytotoxicity via actin cytoskeleton modification (36, 37)<br>- Adhesion to host epithelial cells, hemagglutination, biofilm formation (3)	*Escherichia coli subtype APEC*	αβ	AAO21903.1
**Hap**	**3**	- Serine protease, autoproteolytic release from the OM (38)<br>- Adhesion to host ECM (39)<br>- Bacterial aggregation via self-association (40)	*Haemophilus influenzae*	αβ	AAB03707.1
**MspA (AusI)**	**3**	- Serine protease, autoproteolytic release from the OM (41)<br>- Adhesion to host epithelial cells (41)<br>- Receptor-mediated host cell entry, nuclear localisation, apoptosis via histone degradation (42)	*Neisseria meningitidis*	αβ	AAF42325.1
**IgA1 protease**	**3**	- Serine protease, autoproteolytic release from the OM, immune evasion via IgA1 degradation (43)	*Haemophilus influenzae strain KW20*	αβ	AAC22651.1
**IgA1 protease**	**3**	- Serine protease, autoproteolytic release from the OM, immune evasion via IgA1 degradation (44)	*Haemophilus influenzae strain HK61*	αβ	AAA24968.1
**IgA1 protease**	**3**	- Serine protease, autoproteolytic release from the OM, immune evasion via IgA1 degradation (45)<br>- Intracellular invasion and persistence (46-48)	*Neisseria gonorrhoeae*	αβ	CAA28538.1
**App**	**3**	- Serine protease, autoproteolytic release from the OM (49)<br>- Adhesion to host epithelial cells (49)<br>- Receptor-mediated host cell entry, nuclear localisation, apoptosis promotion via histone degradation (42)	*Neisseria meningitidis*	αβ	AAQ08950.1
**RadD**	**4**	- Multi-species biofilm formation (with streptococcal strains) and lymphocyte agglutination (50)<br>- Induction of lymphocyte cell death (51)	*Fusobacterium nucleatum*	β	AAL93652.1
**Ag43**	**4**	- Bacterial aggregation and biofilm formation via self-association (52, 53)<br>- Adhesion to host cells (54)	*Escherichia coli strain CFT073*	β	AAN79747.1
**Cah**	**4**	- Bacterial aggregation and biofilm formation via self-association, calcium binding (55)	*Escherichia coli subtype EHEC*	β	AAG55356.1
**AIDA-I**	**4**	- Bacterial aggregation and biofilm formation via self-association (56)<br>- Adhesion to host epithelial cells (57)	*Diarrhoeagenic Escherichia coli strains*	β	CAA46156.1
**YapC**	**4**	- Bacterial aggregation, biofilm formation, adhesion to host epithelial and macrophage cells (58)	*Yersinia pestis*	β	CAC14222.1
**TibA**	**4**	- Bacterial aggregation and biofilm formation via self-association (59)<br>- Adhesion to and invasion of host epithelial cells (60)	*Escherichia coli subtype ETEC*	β	AAD41751.1
**YapA**	**4**	- Homologous to host adhesins, but does not contribute to bacterial aggregation or hemagglutination (61)	*Yersinia pestis*	α β	AKB89180.1
**YcgV**	**4**	- Adhesion to abiotic surfaces, biofilm formation (62)	*Escherichia coli strain K-12*	β	AYG19712.1
**YpjA (EhaD)**	**4**	- Adhesion to abiotic surfaces (62)<br>- Biofilm formation (63)	*Escherichia coli subtype EHEC*	β	AYG18348.1
**rOmpA (Sca0)**	**5**	- Adhesion to and invasion of host endothelial cells by interacting with $\alpha 2\beta 1$ integrin (64)<br>- Adhesion to host fibroblast cells (65)	*Rickettsia rickettsii*	β	AAA26380.1
**ScaA**	**5**	- Adhesion to host epithelial cells (66)	*Orientia tsutsugamushi*	β	AJC11311.1
**VacA**	**5**	- Intracellular vacuolating cytotoxicity (67) involving apoptosis via mitochondrial damage (68)	*Helicobacter pylori*	β	AAA17657.1
**PLB**	**6**	- GDSL-lipase, phospholipase B activity (69)	*Moraxella bovis*	α	AAK53448.1
**EstA**	**6**	- GDSL-lipase, activity promotes rhamnolipid production, cell motility, and high biofilm surface coverage (70)	*Pseudomonas aeruginosa*	α	AAB61674.1
**McaP**	**6**	- GDSL-lipase, phospholipase B activity, adhesion to epithelial cells (71, 72)	*Moraxella catarrhalis*	α	RKM05276.1

**ApeE**	**6**	- GDSL-lipase, hydrolysis of fatty acid naphthyl and p-nitrophenyl esters (73)	*Salmonella enterica Typhimurium*[d]	α	AAC38796.1
**Pmp6**	**7**	- Host cell adhesion and entry (74, 75)	*Chlamydia pneumoniae*	β	AAD18588.1
**PmpA**	**7**	- Host cell adhesion and entry (75)	*Chlamydia trachomatis*	β	AAC68009.1
**PmpE**	**7**	- Host cell adhesion and entry (75)	*Chlamydia trachomatis*	β	AAC68467.1
**PmpF**	**7**	- Host cell adhesion and entry (75)	*Chlamydia trachomatis*	β	AAC68468.1
**PmpG**	**7**	- Host cell adhesion and entry (75)	*Chlamydia trachomatis*	β	AAC68469.1
**PmpH**	**7**	- Host cell adhesion and entry (75)	*Chlamydia trachomatis*	β	AAC68470.1
**Pmp21**	**7**	- Host cell adhesion and entry (74, 75) via EGFR binding (76)<br>- Formation of oligomers (77)	*Chlamydia pneumoniae*	β	AAD19099.1
**PmpD**	**7**	- Host cell adhesion and entry (78)<br>- Formation of oligomers and higher-order ring structures (79, 80)	*Chlamydia trachomatis*	β	AAC68408.1
**PmpI**	**7**	- Host cell adhesion and entry (75)	*Chlamydia trachomatis*	β	AAC68472.1
**AatA**	**8**	- Bacterial aggregation (81)<br>- Adhesion to chicken fibroblast cells (82, 83)<br>- Deletion reduced tissue damage and mortality of infected chickens (83)	*Escherichia coli* (APEC)	β	ADJ53351.1
**ShdA**	**8**	- Adhesion to ECM proteins including fibronectin and collagen I, but NOT collagen IV or antithrombin III (84-86)<br>- Adhesion to connective tissue and basement membrane of murine caecum (84)<br>- Deletion reduced murine intestinal colonisation (87)	*Salmonella enterica Typhimurium*[d]	β	AAD25110.2
**YapE**	**8**	- Bacterial aggregation (88)<br>- Adhesion to human lung epithelial and macrophage-like cells (89) (88)<br>- Deletion reduced dissemination to the lymph nodes and spleen in mice models (88)	*Yersinia pestis*	β	AJI90856.1
**IcsA (VirG)**	**8**	- Bacterial aggregation and biofilm formation in the presence of bile salts (90)<br>- Self-association (91)<br>- Adhesion to human epithelial cells and macrophages (92)<br>- Actin-based intracellular motility (93, 94)<br>- No ECM binding studies published; however, IcsA binds integral membrane proteins of unknown identity from macrophage membrane fractions (95)	*Shigella flexneri*	β	AAA26547.1
**MisL**	**8**	- Bacterial aggregation and biofilm formation (96)<br>- Adhesion to human epithelial cells (96)<br>- Adhesion to ECM proteins including fibronectin and collagen IV, which increased invasion of human colonic epithelial cells (97)	*Salmonella enterica Typhimurium*[d]	β	AAD16954.1
**EhaA**	**8**	- Bacterial aggregation and biofilm formation (63)<br>- Adhesion to bovine epithelial cells (63)<br>- No ECM binding studies published.	*Escherichia coli subtype EHEC*	β	WP_105288411.1
**EhaJ**	**8**	- Biofilm formation, which increases upon glycosylation of the passenger domain (98)<br>- Adhesion to ECM proteins including collagen I, collagen II, collagen III, collagen V, fibronectin, fibrinogen, and laminin (98)	*Escherichia coli subtype EPEC*	β	WP_137526983.1
**UpaE**	**8**	- Biofilm formation (99)<br>- Adhesion to ECM proteins including fibronectin, laminin, and collagens I, II, and V (99)	*Escherichia coli subtype UPEC*	β	WP_012311963.1
**YfaL (EhaC)**	**8**	- Adhesion to abiotic surfaces, biofilm formation (62)	*Escherichia coli strain K-12*	β	AYG18743.1

**YapG**	**8**	- Homologous to host adhesins, but does not contribute to bacterial aggregation or hemagglutination (61)	*Yersinia pseudotuberculosis*	β	VEG86139.1
**BapC**	**9**	- Adhesion to host epithelial cells (100),<br>- Serum resistance, possibly involving complement inhibition (101, 102)	*Bordetella pertussis*	β	CAC14167.1
**Prn (pertactin)**	**9**	- Adhesion to mammalian host cells via RGD motif (103)<br>- Evasion of neutrophil-mediated clearance (innate immune response) through an unknown mechanism that does not involve the RGD (104)<br>- Evasion of inflammatory immune response (105)	*Bordetella pertussis*	β	AAA22980.1
**Vag8**	**9**	- Serum resistance via inhibiting C1-INH, a complement system regulator (106, 107)<br>- Adhesion to human respiratory epithelial cells (108)	*Bordetella pertussis*	β	AAC31247.1
**BrkA**	**9**	- Serum resistance via complement inhibition (109)<br>- Adhesion to human respiratory epithelial cells (108)	*Bordetella pertussis*	β	AAA51646.1
**EhaB**	**10**	- Adhesion to host ECM laminin and collagen I (110)<br>- Biofilm formation but not aggregation (63)	*Escherichia coli (subtype EHEC)*	β	WP_016262982.1
**UpaC**	**10**	- Biofilm formation (111)	*Escherichia coli (subtype UPEC)*	β	CDN80760.1
**ImaA**	**11**	- Regulation of host immune responses through unknown interaction (112, 113)<br>- Increases colonisation (114)	*Helicobacter pylori*	α β	WP_165561904.1
**VlpC**	**11**	- Increases colonisation (114)	*Helicobacter pylori*	α β	AAD07969.1
**FaaA**	**11**	- Increases colonisation, localizes to the flagellar sheath and contributes to motility (114)	*Helicobacter pylori*	α β	AQM65931.1
**UpaB**	**12**	- Adhesion to host ECM fibronectin, fibrinogen, and laminin (111, 115)<br>- Adhesion to host fibroblast cells (116)<br>- Colonisation of the bladder (111)	*Escherichia coli strain CFT073 (UPEC)*	β	AAN78907.1
**UpaH**	**12**	- Adhesion to host ECM collagen V, fibronectin, and laminin but not to collagen IV or fibrinogen (117)<br>- Biofilm formation (118) (117)	*Escherichia coli (subtype UPEC)*	β	PIM45967.1
**Pmp20**	**12**	- Host cell adhesion and entry (74)	*Chlamydia pneumoniae*	β	AAD18680.1
**PmpB**	**12**	- Host cell adhesion and entry (75)<br>-	*Chlamydia trachomatis*	β	AAC68010.1
**PmpC**	**12**	- Host cell adhesion and entry (75)<br>-	*Chlamydia trachomatis*	β	AAC68011.1
**YapJ**	**13**	- Adhesion to host ECM (119)	*Yersinia pestis*	β	AKB89685.1
**YapK**	**13**	- Adhesion to host epithelial cells and ECM (119)	*Yersinia pestis*	β	AJI93318.1
**YapV**	**13**	- Adhesion to host epithelial cells and ECM (119)<br>- Binds N-WASP (120)	*Yersinia pestis*	β	AJI91077.1
**CapA**	**13**	- Adhesion to human epithelial cells and increased colonisation of chicken gut (121)	*Campylobacter jejuni*	β	CAL34774.1
**BmaC**	**13**	- Adhesion to host epithelial cells and ECM (fibronectin) (122)	*Brucella suis*	β	A0A0H3GGE2.1
**BatB**	**13**	- Immune evasion via immunoglobulin binding (123)	*Bordetella bronchiseptica*	β	KAB1575196.1
**rOmpB (Sca5)**	**13**	- Adhesion to and invasion of host epithelial cells (124, 125)<br>- Invasion of host cells via endocytosis after binding the Ku70 receptor on mammalian cell surfaces (124)	*Rickettsia rickettsii*	β	CAA34403.1
**AlpA**	**13**	- Adhesion to epithelial cells (126)	*Helicobacter pylori*	α	CAB05386.1

Supplementary Data Function and classification of Autotransporters

**AoaA**	**13**	- Promotes rhizobial symbiosis in stem nodules via evading plant defenses (127)	*Azorhizobium caulinodans* (plant symbiont)	β	BAF88633.1
**BatA**	**13**	- GDSL-lipase, promotes intracellular survival (128)	*Burkholderia mallei*	α	AAU47766.1
**BapF**	**13**	- Bacterial aggregation (129)	*Bordetella bronchiseptica*	β	WP_01092646 5.1
**XatA**	**13**	- Bacterial aggregation, biofilm formation (130)	*Xylella fastidios a* (plant pathogen)	β	AAO28401.1
**Aae**	**14**	- Adhesion to host epithelial cells (OM-anchored) (131)<br>- Biofilm formation (132)	*Actinobacillus actinomycetemc omitans*	α	AAP21063.1
**Sca1**	**14**	- Adhesion to host epithelial cells (133)	*Rickettsia rickettsii*	α	AAU06432.1
**Sca2**	**14**	- Adhesion to and invasion of host endothelial cells (134)<br>- Actin-based intracellular motility (135)	*Rickettsia rickettsii*	α	ABV75720.1
**ScaC**	**14**	- Adhesion to host epithelial cells and ECM (136)	*Orientia tsutsugamushi*	α	SPR10606.1
**SSP**	**15**	- Subtilisin-like (137)	*Serratia marcescens*	αβ	AAA26572.1
**PrtT**	**15**	- Subtilisin-like (138)	*Serratia marcescens*	αβ	CAA42236.1
**PspA**	**15**	- Subtilisin-like (139)	*Pseudomonas fluorescens*	αβ	BAA36466.1
**PspB_X**	**15**	- Subtilisin-like (140)	*Xylella fastidiosa (plant pathogen)*	αβ	AAO28109.1
**Fusolisin**	**15**	- Subtilisin-like (141)<br>- Degradation of ECM proteins and IgA (142)	*Fusobacterium nucleatum*	αβ	AIC79906.1
**PspB_F**	**15**	- Subtilisin-like, serum resistance (143)	*Pseudomonas fluorescens*	αβ	BAA36467.1
**EprS**	**15**	- Subtilisin-like, immunomodulator (144)<br>- Pleiotropic regulator of bacterial virulence (145)	*Pseudomonas aeruginosa*	αβ	AAG06923.1
**Pta**	**15**	- Subtilisin-like, cytotoxicity, bacterial aggregation, OM-associated (146)	*Proteus mirabilis*	αβ	WP_01236832 5.1
**Pfa1**	**15**	- Subtilisin-like, cytotoxicity, biofilm production, interaction with host cells, and immunomodulation (147)	*Pseudomonas fluorescens*	αβ	ACR15124.1
**SphB1**	**15**	- Subtilisin-like, maturation of filamentous hemagglutinin at the outer membrane (148, 149)<br>- Adhesion to human respiratory epithelial cells (108)<br>-	*Bordetella pertussis*	αβ	CAE40596.1
**AasP**	**15**	- Subtilisin-like, maturation of outer membrane protein OmlA (150)<br>- Biofilm regulation (151)	*Actinobacillus pleuropneumoni ae (causes porcine pneumonia)*	αβ	ABF22613.1
**NalP (AspA)**	**15**	- Subtilisin-like, maturation of virulence factors including App, MspA, and IgA1 protease ATs (152, 153)<br>- Serum resistance via degrading C3 of the complement system (154)<br>- Biofilm regulation (155)<br>- Uptake into endothelial cells that alters metabolism (156)	*Neisseria meningitidis*	αβ	AAN71715.1
**BcaA**	**15**	- Subtilisin-like, invasion of host epithelial cells, dissemination from intranasal to spleen infection (157)	*Burkholderia pseudomallei*	αβ	WP_03874214 6.1
**CapC**	**16**	- Adhesion to host epithelial cells (158)	*Campylobacter jejuni*	β	ABV52877.1
**Fap2**	**16**	- Bacterial aggregation, adhesion to host kidney cells, hemagglutination (159)	*Fusobacterium nucleatum*	β	WP_05922289 8.2

**NanB**	**16**	- Sialidase, nutrient acquisition (160)	*Pasteurella multocida*	β	AAG35309.1
**TcfA**	×	- Adhesion to human respiratory epithelial cells (108)<br>- Deletion decreased 10X the colonisation of murine trachea (161)	*Bordetella pertussis*	C	AZY81973.1
**MapA**	×	- Acid phosphatase (162)	*Moraxella catarrhalis*	αβ	ABP88873.1
**AutA**	×	- Bacterial aggregation (163)<br>- Present in pathogenic and commensal Neisseria species (163)<br>- Interspecies biofilms (164)	*Neisseria meningitidis*	αβ	AHK23078.1
**AutB**	×	- Biofilm formation (165)	*Neisseria meningitidis*	β	AOC61149.1
**AaaA**	×	- Aminopeptidase, nitrogen acquisition via arginine metabolism (166)	*Pseudomonas aeruginosa*	α	AAG03717.1

[a]*G: Phylogenetic group as per Figure 5*

[b]*SS: Passenger domain secondary structure prediction by PSIPRED: α-helical (α), β-strand (β), mix (αβ), or coil (C)*

[c]*ID: GenBank accession number*

[d]*Salmonella enterica subs. enterica serovar* **Typhimurium**

ECM: Extracellular matrix

Referecencs
1. Mitchell AL, Attwood TK, Babbitt PC, Blum M, Bork P, Bridge A, et al. InterPro in 2019: improving coverage, classification and access to protein sequence annotations. *Nucleic Acids Res* (2019) 47(D1):D351-D60. doi: 10.1093/nar/gky1100.
2. Jones DT. Protein secondary structure prediction based on position-specific scoring matrices. *J Mol Biol* (1999) 292(2):195-202. doi: 10.1006/jmbi.1999.3091.
3. Habouria H, Pokharel P, Maris S, Garenaux A, Bessaiah H, Houle S, et al. Three new serine-protease autotransporters of Enterobacteriaceae (SPATEs) from extra-intestinal pathogenic Escherichia coli and combined role of SPATEs for cytotoxicity and colonization of the mouse kidney. *Virulence* (2019) 10(1):568-87. doi: 10.1080/21505594.2019.1624102.
4. Pokharel P, Díaz JM, Bessaiah H, Houle S, Guerrero-Barrera AL, Dozois CM. The Serine Protease Autotransporters TagB, TagC, and Sha from Extraintestinal Pathogenic Escherichia coli Are Internalized by Human Bladder Epithelial Cells and Cause Actin Cytoskeletal Disruption. (2020) 21(9):3047.
5. Al-Hasani K, Henderson IR, Sakellaris H, Rajakumar K, Grant T, Nataro JP, et al. The sigA gene which is borne on the she pathogenicity island of Shigella flexneri 2a encodes an exported cytopathic protease involved in intestinal fluid accumulation. *Infect Immun* (2000) 68(5):2457-63. doi: 10.1128/iai.68.5.2457-2463.2000.
6. Al-Hasani K, Navarro-Garcia F, Huerta J, Sakellaris H, Adler B. The immunogenic SigA enterotoxin of Shigella flexneri 2a binds to HEp-2 cells and induces fodrin redistribution in intoxicated epithelial cells. *PLoS One* (2009) 4(12):e8223. doi: 10.1371/journal.pone.0008223.
7. Drago-Serrano ME, Parra SG, Manjarrez-Hernandez HA. EspC, an autotransporter protein secreted by enteropathogenic Escherichia coli (EPEC), displays protease activity on human hemoglobin. *FEMS Microbiol Lett* (2006) 265(1):35-40. doi: 10.1111/j.1574-6968.2006.00463.x.
8. Vidal JE, Navarro-Garcia F. EspC translocation into epithelial cells by enteropathogenic Escherichia coli requires a concerted participation of type V and III secretion systems. *Cell Microbiol* (2008) 10(10):1975-86. doi: 10.1111/j.1462-5822.2008.01181.x.
9. Navarro-Garcia F, Sears C, Eslava C, Cravioto A, Nataro JP. Cytoskeletal effects induced by Pet, the serine protease enterotoxin of enteroaggregative *Escherichia coli*. *Infect Immun* (1999) 67(5):2184-92.
10. Navarro-Garcia F, Canizalez-Roman A, Luna J, Sears C, Nataro JP. Plasmid-encoded toxin of enteroaggregative *Escherichia coli* is internalized by epithelial cells. *Infect Immun* (2001) 69(2):1053-60. doi: 10.1128/IAI.69.2.1053-1060.2001.
11. Djafari S, Ebel F, Deibel C, Kramer S, Hudel M, Chakraborty T. Characterization of an exported protease from Shiga toxin-producing Escherichia coli. *Mol Microbiol* (1997) 25(4):771-84. doi: 10.1046/j.1365-2958.1997.5141874.x.
12. Brunder W, Schmidt H, Karch H. EspP, a novel extracellular serine protease of enterohaemorrhagic Escherichia coli O157:H7 cleaves human coagulation factor V. *Mol Microbiol* (1997) 24(4):767-78. doi: 10.1046/j.1365-2958.1997.3871751.x.
13. Brockmeyer J, Aldick T, Soltwisch J, Zhang W, Tarr PI, Weiss A, et al. Enterohaemorrhagic Escherichia coli haemolysin is cleaved and inactivated by serine protease EspPalpha. *Environ Microbiol* (2011) 13(5):1327-41. doi: 10.1111/j.1462-2920.2011.02431.x.
14. Kuo KH, Khan S, Rand ML, Mian HS, Brnjac E, Sandercock LE, et al. EspP, an Extracellular Serine Protease from Enterohemorrhagic E. coli, Reduces Coagulation Factor Activities, Reduces Clot Strength, and Promotes Clot Lysis. *PLoS One* (2016) 11(3):e0149830. doi: 10.1371/journal.pone.0149830.
15. Guyer DM, Radulovic S, Jones FE, Mobley HL. Sat, the secreted autotransporter toxin of uropathogenic Escherichia coli, is a vacuolating cytotoxin for bladder and kidney epithelial cells. *Infect Immun* (2002) 70(8):4539-46. doi: 10.1128/iai.70.8.4539-4546.2002.
16. Lievin-Le Moal V, Comenge Y, Ruby V, Amsellem R, Nicolas V, Servin AL. Secreted autotransporter toxin (Sat) triggers autophagy in epithelial cells that relies on cell detachment. *Cell Microbiol* (2011) 13(7):992-1013. doi: 10.1111/j.1462-5822.2011.01595.x.

17. Maroncle NM, Sivick KE, Brady R, Stokes FE, Mobley HL. Protease activity, secretion, cell entry, cytotoxicity, and cellular targets of secreted autotransporter toxin of uropathogenic Escherichia coli. *Infect Immun* (2006) 74(11):6124-34. doi: 10.1128/IAI.01086-06.
18. Vieira PCG, Espinoza-Culupu AO, Nepomuceno R, Alves MR, Lebrun I, Elias WP, et al. Secreted autotransporter toxin (Sat) induces cell damage during enteroaggregative Escherichia coli infection. *PLoS One* (2020) 15(2):e0228959. doi: 10.1371/journal.pone.0228959.
19. Leyton DL, Adams LM, Kelly M, Sloan J, Tauschek M, Robins-Browne RM, et al. Contribution of a novel gene, rpeA, encoding a putative autotransporter adhesin to intestinal colonization by rabbit-specific enteropathogenic Escherichia coli. *Infect Immun* (2007) 75(9):4664-9. doi: 10.1128/IAI.00972-06.
20. Henderson IR, Czeczulin J, Eslava C, Noriega F, Nataro JP. Characterization of pic, a secreted protease of Shigella flexneri and enteroaggregative Escherichia coli. *Infect Immun* (1999) 67(11):5587-96.
21. Ayala-Lujan JL, Vijayakumar V, Gong M, Smith R, Santiago AE, Ruiz-Perez F. Broad spectrum activity of a lectin-like bacterial serine protease family on human leukocytes. *PLoS One* (2014) 9(9):e107920. doi: 10.1371/journal.pone.0107920.
22. Abreu AG, Fraga TR, Granados Martinez AP, Kondo MY, Juliano MA, Juliano L, et al. The Serine Protease Pic From Enteroaggregative Escherichia coli Mediates Immune Evasion by the Direct Cleavage of Complement Proteins. *J Infect Dis* (2015) 212(1):106-15. doi: 10.1093/infdis/jiv013.
23. Parham NJ, Srinivasan U, Desvaux M, Foxman B, Marrs CF, Henderson IR. PicU, a second serine protease autotransporter of uropathogenic Escherichia coli. *FEMS Microbiol Lett* (2004) 230(1):73-83. doi: 10.1016/S0378-1097(03)00862-0.
24. Bhullar K, Zarepour M, Yu H, Yang H, Croxen M, Stahl M, et al. The Serine Protease Autotransporter Pic Modulates Citrobacter rodentium Pathogenesis and Its Innate Recognition by the Host. *Infect Immun* (2015) 83(7):2636-50. doi: 10.1128/IAI.00025-15.
25. Dutta PR, Cappello R, Navarro-Garcia F, Nataro JP. Functional comparison of serine protease autotransporters of Enterobacteriaceae. *Infect Immun* (2002) 70(12):7105-13. doi: 10.1128/iai.70.12.7105-7113.2002.
26. Otto BR, van Dooren SJ, Nuijens JH, Luirink J, Oudega B. Characterization of a hemoglobin protease secreted by the pathogenic Escherichia coli strain EB1. *J Exp Med* (1998) 188(6):1091-103. doi: 10.1084/jem.188.6.1091.
27. Provence DL, Curtiss R, 3rd. Isolation and characterization of a gene involved in hemagglutination by an avian pathogenic Escherichia coli strain. *Infect Immun* (1994) 62(4):1369-80.
28. Kostakioti M, Stathopoulos C. Functional analysis of the Tsh autotransporter from an avian pathogenic Escherichia coli strain. *Infect Immun* (2004) 72(10):5548-54. doi: 10.1128/IAI.72.10.5548-5554.2004.
29. Hart E, Yang J, Tauschek M, Kelly M, Wakefield MJ, Frankel G, et al. RegA, an AraC-like protein, is a global transcriptional regulator that controls virulence gene expression in Citrobacter rodentium. *Infect Immun* (2008) 76(11):5247-56. doi: 10.1128/IAI.00770-08.
30. Gutierrez D, Pardo M, Montero D, Onate A, Farfan MJ, Ruiz-Perez F, et al. TleA, a Tsh-like autotransporter identified in a human enterotoxigenic Escherichia coli strain. *Infect Immun* (2015) 83(5):1893-903. doi: 10.1128/IAI.02976-14.
31. Kumar P, Luo Q, Vickers TJ, Sheikh A, Lewis WG, Fleckenstein JM. EatA, an immunogenic protective antigen of enterotoxigenic Escherichia coli, degrades intestinal mucin. *Infect Immun* (2014) 82(2):500-8. doi: 10.1128/IAI.01078-13.
32. Roy K, Kansal R, Bartels SR, Hamilton DJ, Shaaban S, Fleckenstein JM. Adhesin degradation accelerates delivery of heat-labile toxin by enterotoxigenic Escherichia coli. *J Biol Chem* (2011) 286(34):29771-9. doi: 10.1074/jbc.M111.251546.
33. Leyton DL, Sloan J, Hill RE, Doughty S, Hartland EL. Transfer region of pO113 from enterohemorrhagic Escherichia coli: similarity with R64 and identification of a novel plasmid-encoded autotransporter, EpeA. *Infect Immun* (2003) 71(11):6307-19. doi: 10.1128/iai.71.11.6307-6319.2003.
34. Maldonado-Contreras A, Birtley JR, Boll E, Zhao Y, Mumy KL, Toscano J, et al. Shigella depends on SepA to destabilize the intestinal epithelial integrity via cofilin activation. *Gut Microbes* (2017) 8(6):544-60. doi: 10.1080/19490976.2017.1339006.

35. Schmidt H, Zhang WL, Hemmrich U, Jelacic S, Brunder W, Tarr PI, et al. Identification and characterization of a novel genomic island integrated at selC in locus of enterocyte effacement-negative, Shiga toxin-producing Escherichia coli. *Infect Immun* (2001) 69(11):6863-73. doi: 10.1128/IAI.69.11.6863-6873.2001.
36. Parreira VR, Gyles CL. A novel pathogenicity island integrated adjacent to the thrW tRNA gene of avian pathogenic Escherichia coli encodes a vacuolating autotransporter toxin. *Infect Immun* (2003) 71(9):5087-96. doi: 10.1128/iai.71.9.5087-5096.2003.
37. Diaz JM, Dozois CM, Avelar-Gonzalez FJ, Hernandez-Cuellar E, Pokharel P, de Santiago AS, et al. The vacuolating autotransporter toxin (Vat) of *Escherichia coli* causes cell cytoskeleton changes and produces non-lysosomal vacuole formation in bladder epithelial cells. *Front Cell Infect Microbiol* (2020) 10:299. doi: 10.3389/fcimb.2020.00299.
38. Fink DL, Cope LD, Hansen EJ, Geme JW, 3rd. The Hemophilus influenzae Hap autotransporter is a chymotrypsin clan serine protease and undergoes autoproteolysis via an intermolecular mechanism. *J Biol Chem* (2001) 276(42):39492-500. doi: 10.1074/jbc.M106913200.
39. Fink DL, Buscher AZ, Green B, Fernsten P, St Geme JW, 3rd. The Haemophilus influenzae Hap autotransporter mediates microcolony formation and adherence to epithelial cells and extracellular matrix via binding regions in the C-terminal end of the passenger domain. *Cell Microbiol* (2003) 5(3):175-86. doi: 10.1046/j.1462-5822.2003.00266.x.
40. Meng G, Spahich N, Kenjale R, Waksman G, St Geme JW, 3rd. Crystal structure of the *Haemophilus influenzae* Hap adhesin reveals an intercellular oligomerization mechanism for bacterial aggregation. *EMBO J* (2011) 30(18):3864-74. doi: 10.1038/emboj.2011.279.
41. Turner DP, Marietou AG, Johnston L, Ho KK, Rogers AJ, Wooldridge KG, et al. Characterization of MspA, an immunogenic autotransporter protein that mediates adhesion to epithelial and endothelial cells in Neisseria meningitidis. *Infect Immun* (2006) 74(5):2957-64. doi: 10.1128/IAI.74.5.2957-2964.2006.
42. Khairalla AS, Omer SA, Mahdavi J, Aslam A, Dufailu OA, Self T, et al. Nuclear trafficking, histone cleavage and induction of apoptosis by the meningococcal App and MspA autotransporters. *Cell Microbiol* (2015) 17(7):1008-20. doi: 10.1111/cmi.12417.
43. Poulsen K, Brandt J, Hjorth JP, Thogersen HC, Kilian M. Cloning and sequencing of the immunoglobulin A1 protease gene (iga) of Haemophilus influenzae serotype b. *Infect Immun* (1989) 57(10):3097-105.
44. Poulsen K, Reinholdt J, Kilian M. A comparative genetic study of serologically distinct Haemophilus influenzae type 1 immunoglobulin A1 proteases. *J Bacteriol* (1992) 174(9):2913-21. doi: 10.1128/jb.174.9.2913-2921.1992.
45. Plaut AG, Gilbert JV, Artenstein MS, Capra JD. Neisseria gonorrhoeae and neisseria meningitidis: extracellular enzyme cleaves human immunoglobulin A. *Science* (1975) 190(4219):1103-5. doi: 10.1126/science.810892.
46. Clementi CF, Hakansson AP, Murphy TF. Internalization and trafficking of nontypeable Haemophilus influenzae in human respiratory epithelial cells and roles of IgA1 proteases for optimal invasion and persistence. *Infect Immun* (2014) 82(1):433-44. doi: 10.1128/IAI.00864-13.
47. Senior BW, Stewart WW, Galloway C, Kerr MA. Cleavage of the hormone human chorionic gonadotropin, by the Type 1 IgA1 protease of Neisseria gonorrhoeae, and its implications. *J Infect Dis* (2001) 184(7):922-5. doi: 10.1086/323397.
48. Besbes A, Le Goff S, Antunes A, Terrade A, Hong E, Giorgini D, et al. Hyperinvasive Meningococci Induce Intra-nuclear Cleavage of the NF-kappaB Protein p65/RelA by Meningococcal IgA Protease. *PLoS Pathog* (2015) 11(8):e1005078. doi: 10.1371/journal.ppat.1005078.
49. Serruto D, Adu-Bobie J, Scarselli M, Veggi D, Pizza M, Rappuoli R, et al. Neisseria meningitidis App, a new adhesin with autocatalytic serine protease activity. *Mol Microbiol* (2003) 48(2):323-34. doi: 10.1046/j.1365-2958.2003.03420.x.
50. Kaplan CW, Lux R, Haake SK, Shi W. The Fusobacterium nucleatum outer membrane protein RadD is an arginine-inhibitable adhesin required for inter-species adherence and the structured architecture of multispecies biofilm. *Mol Microbiol* (2009) 71(1):35-47. doi: 10.1111/j.1365-2958.2008.06503.x.

51. Kaplan CW, Ma X, Paranjpe A, Jewett A, Lux R, Kinder-Haake S, et al. Fusobacterium nucleatum outer membrane proteins Fap2 and RadD induce cell death in human lymphocytes. *Infect Immun* (2010) 78(11):4773-8. doi: 10.1128/IAI.00567-10.
52. Heras B, Totsika M, Peters KM, Paxman JJ, Gee CL, Jarrott RJ, et al. The antigen 43 structure reveals a molecular Velcro-like mechanism of autotransporter-mediated bacterial clumping. *Proc Natl Acad Sci USA* (2014) 111(1):457-62. doi: 10.1073/pnas.1311592111.
53. Danese PN, Pratt LA, Dove SL, Kolter R. The outer membrane protein, antigen 43, mediates cell-to-cell interactions within *Escherichia coli* biofilms. *Mol Microbiol* (2000) 37(2):424-32. doi: 10.1046/j.1365-2958.2000.02008.x.
54. Sherlock O, Dobrindt U, Jensen JB, Munk Vejborg R, Klemm P. Glycosylation of the self-recognizing *Escherichia coli* Ag43 autotransporter protein. *J Bacteriol* (2006) 188(5):1798-807. doi: 10.1128/JB.188.5.1798-1807.2006.
55. Torres AG, Perna NT, Burland V, Ruknudin A, Blattner FR, Kaper JB. Characterization of Cah, a calcium-binding and heat-extractable autotransporter protein of enterohaemorrhagic *Escherichia coli*. *Mol Microbiol* (2002) 45(4):951-66. doi: 10.1046/j.1365-2958.2002.03094.x.
56. Sherlock O, Schembri MA, Reisner A, Klemm P. Novel roles for the AIDA adhesin from diarrheagenic *Escherichia coli*: cell aggregation and biofilm formation. *J Bacteriol* (2004) 186(23):8058-65. doi: 10.1128/JB.186.23.8058-8065.2004.
57. Laarmann S, Schmidt MA. The *Escherichia coli* AIDA autotransporter adhesin recognizes an integral membrane glycoprotein as receptor. *Microbiology (Reading)* (2003) 149(Pt 7):1871-82. doi: 10.1099/mic.0.26264-0.
58. Felek S, Lawrenz MB, Krukonis ES. The *Yersinia pestis* autotransporter YapC mediates host cell binding, autoaggregation and biofilm formation. *Microbiology (Reading)* (2008) 154(Pt 6):1802-12. doi: 10.1099/mic.0.2007/010918-0.
59. Sherlock O, Vejborg RM, Klemm P. The TibA adhesin/invasin from enterotoxigenic *Escherichia coli* is self recognizing and induces bacterial aggregation and biofilm formation. *Infect Immun* (2005) 73(4):1954-63. doi: 10.1128/IAI.73.4.1954-1963.2005.
60. Elsinghorst EA, Weitz JA. Epithelial cell invasion and adherence directed by the enterotoxigenic *Escherichia coli* tib locus is associated with a 104-kilodalton outer membrane protein. *Infect Immun* (1994) 62(8):3463-71.
61. Yen YT, Karkal A, Bhattacharya M, Fernandez RC, Stathopoulos C. Identification and characterization of autotransporter proteins of *Yersinia pestis* KIM. *Mol Membr Biol* (2007) 24(1):28-40. doi: 10.1080/09687860600927626.
62. Roux A, Beloin C, Ghigo JM. Combined inactivation and expression strategy to study gene function under physiological conditions: application to identification of new *Escherichia coli* adhesins. *J Bacteriol* (2005) 187(3):1001-13. doi: 10.1128/JB.187.3.1001-1013.2005.
63. Wells TJ, Sherlock O, Rivas L, Mahajan A, Beatson SA, Torpdahl M, et al. EhaA is a novel autotransporter protein of enterohemorrhagic *Escherichia coli* O157:H7 that contributes to adhesion and biofilm formation. *Environ Microbiol* (2008) 10(3):589-604. doi: 10.1111/j.1462-2920.2007.01479.x.
64. Hillman RD, Jr., Baktash YM, Martinez JJ. OmpA-mediated rickettsial adherence to and invasion of human endothelial cells is dependent upon interaction with α2β1 integrin. *Cell Microbiol* (2013) 15(5):727-41. doi: 10.1111/cmi.12068.
65. Li H, Walker DH. rOmpA is a critical protein for the adhesion of *Rickettsia rickettsii* to host cells. *Microb Pathog* (1998) 24(5):289-98. doi: 10.1006/mpat.1997.0197.
66. Ha NY, Sharma P, Kim G, Kim Y, Min CK, Choi MS, et al. Immunization with an autotransporter protein of Orientia tsutsugamushi provides protective immunity against scrub typhus. *PLoS Negl Trop Dis* (2015) 9(3):e0003585. doi: 10.1371/journal.pntd.0003585.
67. Papini E, de Bernard M, Milia E, Bugnoli M, Zerial M, Rappuoli R, et al. Cellular vacuoles induced by Helicobacter pylori originate from late endosomal compartments. *Proc Natl Acad Sci U S A* (1994) 91(21):9720-4. doi: 10.1073/pnas.91.21.9720.
68. Kimura M, Goto S, Wada A, Yahiro K, Niidome T, Hatakeyama T, et al. Vacuolating cytotoxin purified from Helicobacter pylori causes mitochondrial damage in human gastric cells. *Microb Pathog* (1999) 26(1):45-52. doi: 10.1006/mpat.1998.0241.

69. Farn JL, Strugnell RA, Hoyne PA, Michalski WP, Tennent JM. Molecular characterization of a secreted enzyme with phospholipase B activity from Moraxella bovis. *J Bacteriol* (2001) 183(22):6717-20. doi: 10.1128/JB.183.22.6717-6720.2001.
70. Wilhelm S, Gdynia A, Tielen P, Rosenau F, Jaeger KE. The autotransporter esterase EstA of Pseudomonas aeruginosa is required for rhamnolipid production, cell motility, and biofilm formation. *J Bacteriol* (2007) 189(18):6695-703. doi: 10.1128/JB.00023-07.
71. Timpe JM, Holm MM, Vanlerberg SL, Basrur V, Lafontaine ER. Identification of a *Moraxella catarrhalis* outer membrane protein exhibiting both adhesin and lipolytic activities. *Infect Immun* (2003) 71(8):4341-50. doi: 10.1128/iai.71.8.4341-4350.2003.
72. Lipski SL, Akimana C, Timpe JM, Wooten RM, Lafontaine ER. The *Moraxella catarrhalis* autotransporter McaP is a conserved surface protein that mediates adherence to human epithelial cells through its N-terminal passenger domain. *Infect Immun* (2007) 75(1):314-24. doi: 10.1128/IAI.01330-06.
73. Carinato ME, Collin-Osdoby P, Yang X, Knox TM, Conlin CA, Miller CG. The apeE gene of *Salmonella typhimurium* encodes an outer membrane esterase not present in *Escherichia coli*. *J Bacteriol* (1998) 180(14):3517-21. doi: 10.1128/JB.180.14.3517-3521.1998.
74. Molleken K, Schmidt E, Hegemann JH. Members of the Pmp protein family of *Chlamydia pneumoniae* mediate adhesion to human cells via short repetitive peptide motifs. *Mol Microbiol* (2010) 78(4):1004-17. doi: 10.1111/j.1365-2958.2010.07386.x.
75. Becker E, Hegemann JH. All subtypes of the Pmp adhesin family are implicated in chlamydial virulence and show species-specific function. *Microbiologyopen* (2014) 3(4):544-56. doi: 10.1002/mbo3.186.
76. Molleken K, Becker E, Hegemann JH. The *Chlamydia pneumoniae* invasin protein Pmp21 recruits the EGF receptor for host cell entry. *PLoS Pathog* (2013) 9(4):e1003325. doi: 10.1371/journal.ppat.1003325.
77. Luczak SE, Smits SH, Decker C, Nagel-Steger L, Schmitt L, Hegemann JH. The *Chlamydia pneumoniae* adhesin Pmp21 forms oligomers with adhesive properties. *J Biol Chem* (2016) 291(43):22806-18. doi: 10.1074/jbc.M116.728915.
78. Wehrl W, Brinkmann V, Jungblut PR, Meyer TF, Szczepek AJ. From the inside out--processing of the Chlamydial autotransporter PmpD and its role in bacterial adhesion and activation of human host cells. *Mol Microbiol* (2004) 51(2):319-34. doi: 10.1046/j.1365-2958.2003.03838.x.
79. Paes W, Dowle A, Coldwell J, Leech A, Ganderton T, Brzozowski A. The *Chlamydia trachomatis* PmpD adhesin forms higher order structures through disulphide-mediated covalent interactions. *PLoS One* (2018) 13(6):e0198662. doi: 10.1371/journal.pone.0198662.
80. Swanson KA, Taylor LD, Frank SD, Sturdevant GL, Fischer ER, Carlson JH, et al. *Chlamydia trachomatis* polymorphic membrane protein D is an oligomeric autotransporter with a higher-order structure. *Infect Immun* (2009) 77(1):508-16. doi: 10.1128/IAI.01173-08.
81. Wang S, Xia Y, Dai J, Shi Z, Kou Y, Li H, et al. Novel roles for autotransporter adhesin AatA of avian pathogenic *Escherichia coli*: colonization during infection and cell aggregation. *FEMS Immunol Med Microbiol* (2011) 63(3):328-38. doi: 10.1111/j.1574-695X.2011.00862.x.
82. Dai J, Wang S, Guerlebeck D, Laturnus C, Guenther S, Shi Z, et al. Suppression subtractive hybridization identifies an autotransporter adhesin gene of *E. coli* IMT5155 specifically associated with avian pathogenic *Escherichia coli* (APEC). *BMC Microbiol* (2010) 10:236. doi: 10.1186/1471-2180-10-236.
83. Li G, Feng Y, Kariyawasam S, Tivendale KA, Wannemuehler Y, Zhou F, et al. AatA is a novel autotransporter and virulence factor of avian pathogenic *Escherichia coli*. *Infect Immun* (2010) 78(3):898-906. doi: 10.1128/IAI.00513-09.
84. Kingsley RA, Santos RL, Keestra AM, Adams LG, Baumler AJ. Salmonella enterica serotype Typhimurium ShdA is an outer membrane fibronectin-binding protein that is expressed in the intestine. *Mol Microbiol* (2002) 43(4):895-905. doi: 10.1046/j.1365-2958.2002.02805.x.
85. Kingsley RA, Abi Ghanem D, Puebla-Osorio N, Keestra AM, Berghman L, Baumler AJ. Fibronectin binding to the *Salmonella enterica* serotype Typhimurium ShdA autotransporter protein is inhibited by a monoclonal antibody recognizing the A3 repeat. *J Bacteriol* (2004) 186(15):4931-9. doi: 10.1128/JB.186.15.4931-4939.2004.

86. Kingsley RA, Keestra AM, de Zoete MR, Baumler AJ. The ShdA adhesin binds to the cationic cradle of the fibronectin 13FnIII repeat module: evidence for molecular mimicry of heparin binding. *Mol Microbiol* (2004) 52(2):345-55. doi: 10.1111/j.1365-2958.2004.03995.x.
87. Kingsley RA, Humphries AD, Weening EH, De Zoete MR, Winter S, Papaconstantinopoulou A, et al. Molecular and phenotypic analysis of the CS54 island of Salmonella enterica serotype typhimurium: identification of intestinal colonization and persistence determinants. *Infect Immun* (2003) 71(2):629-40. doi: 10.1128/iai.71.2.629-640.2003.
88. Lawrenz MB, Lenz JD, Miller VL. A novel autotransporter adhesin is required for efficient colonization during bubonic plague. *Infect Immun* (2009) 77(1):317-26. doi: 10.1128/IAI.01206-08.
89. Lawrenz MB, Pennington J, Miller VL. Acquisition of omptin reveals cryptic virulence function of autotransporter YapE in *Yersinia pestis*. *Mol Microbiol* (2013) 89(2):276-87. doi: 10.1111/mmi.12273.
90. Koseoglu VK, Hall CP, Rodriguez-Lopez EM, Agaisse H. The autotransporter IcsA promotes *Shigella flexneri* biofilm formation in the presence of bile salts. *Infect Immun* (2019) 87(7). doi: 10.1128/IAI.00861-18.
91. May KL, Grabowicz M, Polyak SW, Morona R. Self-association of the *Shigella flexneri* IcsA autotransporter protein. *Microbiology (Reading)* (2012) 158(Pt 7):1874-83. doi: 10.1099/mic.0.056465-0.
92. Brotcke Zumsteg A, Goosmann C, Brinkmann V, Morona R, Zychlinsky A. IcsA is a *Shigella flexneri* adhesin regulated by the type III secretion system and required for pathogenesis. *Cell Host Microbe* (2014) 15(4):435-45. doi: 10.1016/j.chom.2014.03.001.
93. Mauricio RP, Jeffries CM, Svergun DI, Deane JE. The Shigella Virulence Factor IcsA Relieves N-WASP Autoinhibition by Displacing the Verprolin Homology/Cofilin/Acidic (VCA) Domain. *J Biol Chem* (2017) 292(1):134-45. doi: 10.1074/jbc.M116.758003.
94. Goldberg MB, Theriot JA. *Shigella flexneri* surface protein IcsA is sufficient to direct actin-based motility. *Proc Natl Acad Sci U S A* (1995) 92(14):6572-6. doi: 10.1073/pnas.92.14.6572.
95. Qin J, Doyle MT, Tran ENH, Morona R. The virulence domain of Shigella IcsA contains a subregion with specific host cell adhesion function. *PLoS One* (2020) 15(1):e0227425. doi: 10.1371/journal.pone.0227425.
96. Wang S, Yang D, Wu X, Wang Y, Wang D, Tian M, et al. Autotransporter MisL of *Salmonella enterica* serotype Typhimurium facilitates bacterial aggregation and biofilm formation. *FEMS Microbiol Lett* (2018) 365(17). doi: 10.1093/femsle/fny142.
97. Dorsey CW, Laarakker MC, Humphries AD, Weening EH, Baumler AJ. *Salmonella enterica* serotype Typhimurium MisL is an intestinal colonization factor that binds fibronectin. *Mol Microbiol* (2005) 57(1):196-211. doi: 10.1111/j.1365-2958.2005.04666.x.
98. Easton DM, Totsika M, Allsopp LP, Phan MD, Idris A, Wurpel DJ, et al. Characterization of EhaJ, a New Autotransporter Protein from Enterohemorrhagic and Enteropathogenic *Escherichia coli*. *Front Microbiol* (2011) 2:120. doi: 10.3389/fmicb.2011.00120.
99. Battaglioli EJ, Goh KGK, Atruktsang TS, Schwartz K, Schembri MA, Welch RA. Identification and characterization of a phase-variable element that regulates the autotransporter UpaE in uropathogenic *Escherichia coli*. *mBio* (2018) 9(4). doi: 10.1128/mBio.01360-18.
100. Bokhari H, Bilal I, Zafar S. BapC autotransporter protein of *Bordetella pertussis* is an adhesion factor. *J Basic Microbiol* (2012) 52(4):390-6. doi: 10.1002/jobm.201100188.
101. Noofeli M, Bokhari H, Blackburn P, Roberts M, Coote JG, Parton R. BapC autotransporter protein is a virulence determinant of Bordetella pertussis. *Microb Pathog* (2011) 51(3):169-77. doi: 10.1016/j.micpath.2011.04.004.
102. Riaz MR, Siddiqi AR, Bokhari H. Structural and functional studies of BapC protein of *Bordetella pertussis*. *Microbiol Res* (2015) 174:56-61. doi: 10.1016/j.micres.2015.03.006.
103. Leininger E, Roberts M, Kenimer JG, Charles IG, Fairweather N, Novotny P, et al. Pertactin, an Arg-Gly-Asp-containing Bordetella pertussis surface protein that promotes adherence of mammalian cells. *Proc Natl Acad Sci U S A* (1991) 88(2):345-9. doi: 10.1073/pnas.88.2.345.
104. Inatsuka CS, Xu Q, Vujkovic-Cvijin I, Wong S, Stibitz S, Miller JF, et al. Pertactin is required for Bordetella species to resist neutrophil-mediated clearance. *Infect Immun* (2010) 78(7):2901-9. doi: 10.1128/IAI.00188-10.

105. Hovingh ES, Mariman R, Solans L, Hijdra D, Hamstra HJ, Jongerius I, et al. Bordetella pertussis pertactin knock-out strains reveal immunomodulatory properties of this virulence factor. *Emerg Microbes Infect* (2018) 7(1):39. doi: 10.1038/s41426-018-0039-8.

106. Hovingh ES, van den Broek B, Kuipers B, Pinelli E, Rooijakkers SHM, Jongerius I. Acquisition of C1 inhibitor by *Bordetella pertussis* virulence associated gene 8 results in C2 and C4 consumption away from the bacterial surface. *PLoS Pathog* (2017) 13(7):e1006531. doi: 10.1371/journal.ppat.1006531.

107. Hovingh ES, de Maat S, Cloherty APM, Johnson S, Pinelli E, Maas C, et al. Virulence associated gene 8 of *Bordetella pertussis* enhances contact system activity by inhibiting the regulatory function of complement regulator C1 inhibitor. *Front Immunol* (2018) 9:1172. doi: 10.3389/fimmu.2018.01172.

108. Gasperini G, Biagini M, Arato V, Gianfaldoni C, Vadi A, Norais N, et al. Outer membrane vesicles (OMV)-based and proteomics-driven antigen selection identifies novel factors contributing to *Bordetella pertussis* adhesion to epithelial cells. *Mol Cell Proteomics* (2018) 17(2):205-15. doi: 10.1074/mcp.RA117.000045.

109. Barnes MG, Weiss AA. BrkA protein of *Bordetella pertussis* inhibits the classical pathway of complement after C1 deposition. *Infect Immun* (2001) 69(5):3067-72. doi: 10.1128/IAI.69.5.3067-3072.2001.

110. Wells TJ, McNeilly TN, Totsika M, Mahajan A, Gally DL, Schembri MA. The *Escherichia coli* O157:H7 EhaB autotransporter protein binds to laminin and collagen I and induces a serum IgA response in O157:H7 challenged cattle. *Environ Microbiol* (2009) 11(7):1803-14. doi: 10.1111/j.1462-2920.2009.01905.x.

111. Allsopp LP, Beloin C, Ulett GC, Valle J, Totsika M, Sherlock O, et al. Molecular characterization of UpaB and UpaC, two new autotransporter proteins of uropathogenic *Escherichia coli* CFT073. *Infect Immun* (2012) 80(1):321-32. doi: 10.1128/IAI.05322-11.

112. Sause WE, Castillo AR, Ottemann KM. The Helicobacter pylori autotransporter ImaA (HP0289) modulates the immune response and contributes to host colonization. *Infect Immun* (2012) 80(7):2286-96. doi: 10.1128/IAI.00312-12.

113. Sause WE, Keilberg D, Aboulhouda S, Ottemann KM. The Helicobacter pylori Autotransporter ImaA Tempers the Bacterium's Interaction with alpha5beta1 Integrin. *Infect Immun* (2017) 85(1). doi: 10.1128/IAI.00450-16.

114. Radin JN, Gaddy JA, Gonzalez-Rivera C, Loh JT, Algood HM, Cover TL. Flagellar localization of a Helicobacter pylori autotransporter protein. *mBio* (2013) 4(2):e00613-12. doi: 10.1128/mBio.00613-12.

115. Paxman JJ, Lo AW, Sullivan MJ, Panjikar S, Kuiper M, Whitten AE, et al. Unique structural features of a bacterial autotransporter adhesin suggest mechanisms for interaction with host macromolecules. *Nat Commun* (2019) 10(1):1967. doi: 10.1038/s41467-019-09814-6.

116. Zhu-Ge XK, Pan ZH, Tang F, Mao X, Hu L, Wang SH, et al. The effects of upaB deletion and the double/triple deletion of upaB, aatA, and aatB genes on pathogenicity of avian pathogenic *Escherichia coli*. *Appl Microbiol Biotechnol* (2015) 99(24):10639-54. doi: 10.1007/s00253-015-6925-2.

117. Allsopp LP, Beloin C, Moriel DG, Totsika M, Ghigo JM, Schembri MA. Functional heterogeneity of the UpaH autotransporter protein from uropathogenic *Escherichia coli*. *J Bacteriol* (2012) 194(21):5769-82. doi: 10.1128/JB.01264-12.

118. Allsopp LP, Totsika M, Tree JJ, Ulett GC, Mabbett AN, Wells TJ, et al. UpaH is a newly identified autotransporter protein that contributes to biofilm formation and bladder colonization by uropathogenic *Escherichia coli* CFT073. *Infect Immun* (2010) 78(4):1659-69. doi: 10.1128/IAI.01010-09.

119. Nair MK, De Masi L, Yue M, Galvan EM, Chen H, Wang F, et al. Adhesive properties of YapV and paralogous autotransporter proteins of *Yersinia pestis*. *Infect Immun* (2015) 83(5):1809-19. doi: 10.1128/IAI.00094-15.

120. Besingi RN, Chaney JL, Clark PL. An alternative outer membrane secretion mechanism for an autotransporter protein lacking a C-terminal stable core. *Mol Microbiol* (2013) 90(5):1028-45. doi: 10.1111/mmi.12414.

121. Ashgar SS, Oldfield NJ, Wooldridge KG, Jones MA, Irving GJ, Turner DP, et al. CapA, an autotransporter protein of Campylobacter jejuni, mediates association with human epithelial cells and colonization of the chicken gut. *J Bacteriol* (2007) 189(5):1856-65. doi: 10.1128/JB.01427-06.
122. Posadas DM, Ruiz-Ranwez V, Bonomi HR, Martin FA, Zorreguieta A. BmaC, a novel autotransporter of Brucella suis, is involved in bacterial adhesion to host cells. *Cell Microbiol* (2012) 14(6):965-82. doi: 10.1111/j.1462-5822.2012.01771.x.
123. Williams CL, Haines R, Cotter PA. Serendipitous discovery of an immunoglobulin-binding autotransporter in Bordetella species. *Infect Immun* (2008) 76(7):2966-77. doi: 10.1128/IAI.00323-08.
124. Chan YG, Cardwell MM, Hermanas TM, Uchiyama T, Martinez JJ. Rickettsial outer-membrane protein B (rOmpB) mediates bacterial invasion through Ku70 in an actin, c-Cbl, clathrin and caveolin 2-dependent manner. *Cell Microbiol* (2009) 11(4):629-44. doi: 10.1111/j.1462-5822.2008.01279.x.
125. Uchiyama T, Kawano H, Kusuhara Y. The major outer membrane protein rOmpB of spotted fever group rickettsiae functions in the rickettsial adherence to and invasion of Vero cells. *Microbes Infect* (2006) 8(3):801-9. doi: 10.1016/j.micinf.2005.10.003.
126. Odenbreit S, Till M, Hofreuter D, Faller G, Haas R. Genetic and functional characterization of the alpAB gene locus essential for the adhesion of *Helicobacter pylori* to human gastric tissue. *Mol Microbiol* (1999) 31(5):1537-48. doi: 10.1046/j.1365-2958.1999.01300.x.
127. Suzuki T, Aono T, Liu CT, Suzuki S, Iki T, Yokota K, et al. An outer membrane autotransporter, AoaA, of Azorhizobium caulinodans is required for sustaining high N2-fixing activity of stem nodules. *FEMS Microbiol Lett* (2008) 285(1):16-24. doi: 10.1111/j.1574-6968.2008.01215.x.
128. Lafontaine ER, Chen Z, Huertas-Diaz MC, Dyke JS, Jelesijevic TP, Michel F, et al. The autotransporter protein BatA is a protective antigen against lethal aerosol infection with Burkholderia mallei and Burkholderia pseudomallei. *Vaccine: X* (2019) 1. doi: 10.1016/j.jvacx.2018.100002.
129. Sims P. Biogenesis of BapF : a novel acylated Bordetella autotransporter. Vancouver: The University of British Columbia (2012).
130. Matsumoto A, Huston SL, Killiny N, Igo MM. XatA, an AT-1 autotransporter important for the virulence of *Xylella fastidiosa* Temecula1. *Microbiologyopen* (2012) 1(1):33-45. doi: 10.1002/mbo3.6.
131. Rose JE, Meyer DH, Fives-Taylor PM. Aae, an autotransporter involved in adhesion of *Actinobacillus actinomycetemcomitans* to epithelial cells. *Infect Immun* (2003) 71(5):2384-93. doi: 10.1128/iai.71.5.2384-2393.2003.
132. Nunes AC, Longo PL, Mayer MP. Influence of Aae Autotransporter Protein on Adhesion and Biofilm Formation by Aggregatibacter actinomycetemcomitans. *Braz Dent J* (2016) 27(3):255-60. doi: 10.1590/0103-6440201600260.
133. Riley SP, Goh KC, Hermanas TM, Cardwell MM, Chan YG, Martinez JJ. The *Rickettsia conorii* autotransporter protein Sca1 promotes adherence to nonphagocytic mammalian cells. *Infect Immun* (2010) 78(5):1895-904. doi: 10.1128/IAI.01165-09.
134. Cardwell MM, Martinez JJ. The Sca2 autotransporter protein from *Rickettsia conorii* is sufficient to mediate adherence to and invasion of cultured mammalian cells. *Infect Immun* (2009) 77(12):5272-80. doi: 10.1128/IAI.00201-09.
135. Haglund CM, Choe JE, Skau CT, Kovar DR, Welch MD. Rickettsia Sca2 is a bacterial formin-like mediator of actin-based motility. *Nat Cell Biol* (2010) 12(11):1057-63. doi: 10.1038/ncb2109.
136. Ha NY, Cho NH, Kim YS, Choi MS, Kim IS. An autotransporter protein from *Orientia tsutsugamushi* mediates adherence to nonphagocytic host cells. *Infect Immun* (2011) 79(4):1718-27. doi: 10.1128/IAI.01239-10.
137. Ohnishi Y, Horinouchi S. Extracellular production of a *Serratia marcescens* serine protease in *Escherichia coli*. *Biosci Biotechnol Biochem* (1996) 60(10):1551-8. doi: 10.1271/bbb.60.1551.
138. Miyazaki H, Yanagida N, Horinouchi S, Beppu T. Characterization of the precursor of *Serratia marcescens* serine protease and COOH-terminal processing of the precursor during its excretion through the outer membrane of *Escherichia coli*. *J Bacteriol* (1989) 171(12):6566-72. doi: 10.1128/jb.171.12.6566-6572.1989.

139. Liu L, Chi H, Sun L. Pseudomonas fluorescens: identification of Fur-regulated proteins and evaluation of their contribution to pathogenesis. *Dis Aquat Organ* (2015) 115(1):67-80. doi: 10.3354/dao02874.
140. Chen J, Civerolo E, Tubajika K, Livingston S, Higbee B. Hypervariations of a protease-encoding gene, PD0218 (pspB), in *Xylella fastidiosa* strains causing almond leaf scorch and Pierce's disease in California. *Appl Environ Microbiol* (2008) 74(12):3652-7. doi: 10.1128/AEM.02386-07.
141. Doron L, Coppenhagen-Glazer S, Ibrahim Y, Eini A, Naor R, Rosen G, et al. Identification and characterization of fusolisin, the *Fusobacterium nucleatum* autotransporter serine protease. *PLoS One* (2014) 9(10):e111329. doi: 10.1371/journal.pone.0111329.
142. Bachrach G, Rosen G, Bellalou M, Naor R, Sela MN. Identification of a Fusobacterium nucleatum 65 kDa serine protease. *Oral Microbiol Immunol* (2004) 19(3):155-9. doi: 10.1111/j.0902-0055.2004.00132.x.
143. Sun YY, Sun L. Pseudomonas fluorescens: iron-responsive proteins and their involvement in host infection. *Vet Microbiol* (2015) 176(3-4):309-20. doi: 10.1016/j.vetmic.2015.01.020.
144. Kida Y, Taira J, Yamamoto T, Higashimoto Y, Kuwano K. EprS, an autotransporter protein of *Pseudomonas aeruginosa*, possessing serine protease activity induces inflammatory responses through protease-activated receptors. *Cell Microbiol* (2013) 15(7):1168-81. doi: 10.1111/cmi.12106.
145. Kida Y, Taira J, Kuwano K. EprS, an autotransporter serine protease, plays an important role in various pathogenic phenotypes of Pseudomonas aeruginosa. *Microbiology (Reading)* (2016) 162(2):318-29. doi: 10.1099/mic.0.000228.
146. Alamuri P, Mobley HL. A novel autotransporter of uropathogenic *Proteus mirabilis* is both a cytotoxin and an agglutinin. *Mol Microbiol* (2008) 68(4):997-1017. doi: 10.1111/j.1365-2958.2008.06199.x.
147. Hu YH, Liu CS, Hou JH, Sun L. Identification, characterization, and molecular application of a virulence-associated autotransporter from a pathogenic *Pseudomonas fluorescens* strain. *Appl Environ Microbiol* (2009) 75(13):4333-40. doi: 10.1128/AEM.00159-09.
148. Coutte L, Antoine R, Drobecq H, Locht C, Jacob-Dubuisson F. Subtilisin-like autotransporter serves as maturation protease in a bacterial secretion pathway. *EMBO J* (2001) 20(18):5040-8. doi: 10.1093/emboj/20.18.5040.
149. Coutte L, Willery E, Antoine R, Drobecq H, Locht C, Jacob-Dubuisson F. Surface anchoring of bacterial subtilisin important for maturation function. *Mol Microbiol* (2003) 49(2):529-39. doi: 10.1046/j.1365-2958.2003.03573.x.
150. Ali T, Oldfield NJ, Wooldridge KG, Turner DP, Ala'Aldeen DA. Functional characterization of AasP, a maturation protease autotransporter protein of Actinobacillus pleuropneumoniae. *Infect Immun* (2008) 76(12):5608-14. doi: 10.1128/IAI.00085-08.
151. Tegetmeyer HE, Fricke K, Baltes N. An isogenic Actinobacillus pleuropneumoniae AasP mutant exhibits altered biofilm formation but retains virulence. *Vet Microbiol* (2009) 137(3-4):392-6. doi: 10.1016/j.vetmic.2009.01.026.
152. van Ulsen P, van Alphen L, ten Hove J, Fransen F, van der Ley P, Tommassen J. A Neisserial autotransporter NalP modulating the processing of other autotransporters. *Mol Microbiol* (2003) 50(3):1017-30. doi: 10.1046/j.1365-2958.2003.03773.x.
153. van Ulsen P, Adler B, Fassler P, Gilbert M, van Schilfgaarde M, van der Ley P, et al. A novel phase-variable autotransporter serine protease, AusI, of Neisseria meningitidis. *Microbes Infect* (2006) 8(8):2088-97. doi: 10.1016/j.micinf.2006.03.007.
154. Del Tordello E, Vacca I, Ram S, Rappuoli R, Serruto D. *Neisseria meningitidis* NalP cleaves human complement C3, facilitating degradation of C3b and survival in human serum. *Proc Natl Acad Sci U S A* (2014) 111(1):427-32. doi: 10.1073/pnas.1321556111.
155. Arenas J, Nijland R, Rodriguez FJ, Bosma TN, Tommassen J. Involvement of three meningococcal surface-exposed proteins, the heparin-binding protein NhbA, the alpha-peptide of IgA protease and the autotransporter protease NalP, in initiation of biofilm formation. *Mol Microbiol* (2013) 87(2):254-68. doi: 10.1111/mmi.12097.
156. Dufailu OA, Mahdavi J, Ala'Aldeen DAA, Wooldridge KG, Oldfield NJ. Uptake of Neisserial autotransporter lipoprotein (NalP) promotes an increase in human brain microvascular endothelial cell metabolic activity. *Microb Pathog* (2018) 124:70-5. doi: 10.1016/j.micpath.2018.08.001.

157. Campos CG, Borst L, Cotter PA. Characterization of BcaA, a putative classical autotransporter protein in Burkholderia pseudomallei. *Infect Immun* (2013) 81(4):1121-8. doi: 10.1128/IAI.01453-12.

158. Mehat JW, Park SF, van Vliet AHM, La Ragione RM. CapC, a Novel Autotransporter and Virulence Factor of Campylobacter jejuni. *Appl Environ Microbiol* (2018) 84(16). doi: 10.1128/AEM.01032-18.

159. Coppenhagen-Glazer S, Sol A, Abed J, Naor R, Zhang X, Han YW, et al. Fap2 of Fusobacterium nucleatum is a galactose-inhibitable adhesin involved in coaggregation, cell adhesion, and preterm birth. *Infect Immun* (2015) 83(3):1104-13. doi: 10.1128/IAI.02838-14.

160. Mizan S, Henk A, Stallings A, Maier M, Lee MD. Cloning and characterization of sialidases with 2-6' and 2-3' sialyl lactose specificity from *Pasteurella multocida*. *J Bacteriol* (2000) 182(24):6874-83. doi: 10.1128/jb.182.24.6874-6883.2000.

161. Finn TM, Stevens LA. Tracheal colonization factor: a Bordetella pertussis secreted virulence determinant. *Mol Microbiol* (1995) 16(4):625-34. doi: 10.1111/j.1365-2958.1995.tb02425.x.

162. Hoopman TC, Wang W, Brautigam CA, Sedillo JL, Reilly TJ, Hansen EJ. *Moraxella catarrhalis* synthesizes an autotransporter that is an acid phosphatase. *J Bacteriol* (2008) 190(4):1459-72. doi: 10.1128/JB.01688-07.

163. Arenas J, Cano S, Nijland R, van Dongen V, Rutten L, van der Ende A, et al. The meningococcal autotransporter AutA is implicated in autoaggregation and biofilm formation. *Environ Microbiol* (2015) 17(4):1321-37. doi: 10.1111/1462-2920.12581.

164. Perez-Ortega J, Rodriguez A, Ribes E, Tommassen J, Arenas J. Interstrain Cooperation in Meningococcal Biofilms: Role of Autotransporters NalP and AutA. *Front Microbiol* (2017) 8:434. doi: 10.3389/fmicb.2017.00434.

165. Arenas J, Paganelli FL, Rodriguez-Castano P, Cano-Crespo S, van der Ende A, van Putten JP, et al. Expression of the gene for autotransporter AutB of *Neisseria meningitidis* affects biofilm formation and epithelial transmigration. *Front Cell Infect Microbiol* (2016) 6:162. doi: 10.3389/fcimb.2016.00162.

166. Luckett JC, Darch O, Watters C, Abuoun M, Wright V, Paredes-Osses E, et al. A novel virulence strategy for Pseudomonas aeruginosa mediated by an autotransporter with arginine-specific aminopeptidase activity. *PLoS Pathog* (2012) 8(8):e1002854. doi: 10.1371/journal.ppat.1002854.
